# A comprehensive genomic pan-cancer classification using The Cancer Genome Atlas gene expression data

**DOI:** 10.1186/s12864-017-3906-0

**Published:** 2017-07-03

**Authors:** Yuanyuan Li, Kai Kang, Juno M. Krahn, Nicole Croutwater, Kevin Lee, David M. Umbach, Leping Li

**Affiliations:** 1 Biostatistics and Computational Biology Branch, National Institute of Environmental Health Sciences, NIH, Durham, NC 27709 USA; 2 Genome Integrity & Structural Biology Laboratory, National Institute of Environmental Health Sciences, NIH, Durham, NC 27709 USA

**Keywords:** Pan-cancer, Classification, Ga/KNN, RNA-seq, TCGA, And sex dimorphism

## Abstract

**Background:**

The Cancer Genome Atlas (TCGA) has generated comprehensive molecular profiles. We aim to identify a set of genes whose expression patterns can distinguish diverse tumor types. Those features may serve as biomarkers for tumor diagnosis and drug development.

**Methods:**

Using RNA-seq expression data, we undertook a pan-cancer classification of 9,096 TCGA tumor samples representing 31 tumor types. We randomly assigned 75% of samples into training and 25% into testing, proportionally allocating samples from each tumor type.

**Results:**

We could correctly classify more than 90% of the test set samples. Accuracies were high for all but three of the 31 tumor types, in particular, for READ (rectum adenocarcinoma) which was largely indistinguishable from COAD (colon adenocarcinoma). We also carried out pan-cancer classification, separately for males and females, on 23 sex non-specific tumor types (those unrelated to reproductive organs). Results from these gender-specific analyses largely recapitulated results when gender was ignored. Remarkably, more than 80% of the 100 most discriminative genes selected from each gender separately overlapped. Genes that were differentially expressed between genders included *BNC1*, *FAT2*, *FOXA1*, and *HOXA11*. FOXA1 has been shown to play a role for sexual dimorphism in liver cancer. The differentially discriminative genes we identified might be important for the gender differences in tumor incidence and survival.

**Conclusions:**

We were able to identify many sets of 20 genes that could correctly classify more than 90% of the samples from 31 different tumor types using TCGA RNA-seq data. This accuracy is remarkable given the number of the tumor types and the total number of samples involved. We achieved similar results when we analyzed 23 non-sex-specific tumor types separately for males and females. We regard the frequency with which a gene appeared in those sets as measuring its importance for tumor classification. One third of the 50 most frequently appearing genes were pseudogenes; the degree of enrichment may be indicative of their importance in tumor classification. Lastly, we identified a few genes that might play a role in sexual dimorphism in certain cancers.

**Electronic supplementary material:**

The online version of this article (doi:10.1186/s12864-017-3906-0) contains supplementary material, which is available to authorized users.

## Background

The Cancer Genome Atlas (TCGA) has generated comprehensive molecular profiles including somatic mutation, copy number variation, gene expression, DNA methylation, microRNA expression, and protein expression for more than 30 different human tumor types [1]. Those large datasets provided a great opportunity to examine the global landscape of aberrations at DNA, RNA and protein levels. Pan-cancer analyses have provided comprehensive landscapes of somatic mutations [2–5], somatic copy number alterations [6], mutations in chromatin regulatory factor genes [4], viral expression and host gene fusion [7] in those tumors. Integrated analysis of 12 tumor types using data from gene expression, microRNA expression, protein expression, copy number variation, and DNA methylation revealed genomic features that many tumor types had common as well as features unique particular tumor types [8].

Tumor classifications based on gene expression data have revealed distinct tumor subtypes and uncovered expression patterns that were associated with clinical outcomes [9–14]. Landmark studies like those demonstrated that gene expression data can provide valuable information about tumor characteristics which allow targeted options for treatment and for patient care and management. TCGA RNA-seq gene expression data provides a great opportunity to discover features that can distinguish different tumor types. Those features may serve as biomarkers for tumor diagnosis and/or potential targets for drug development.

Sex differences in cancer susceptibility are one of the most consistent, but least understood, findings in cancer epidemiology [15, 16]. Males are more prone to develop cancer and have worse overall survival than females with the same tumors [17, 18]. For instance, female patients with melanoma tend to exhibit longer survival than male patients [19]. Males have a threefold greater risk for developing bladder cancer than females [20]. Hepatocellular carcinoma, the most common liver cancer, occurs mainly in men. Sex differences in immune response [21] and hormones [22] may play a role. Although additional factors such as sex chromosomes and life style may also contribute, the mechanisms that influence sex differences in cancer susceptibility remain largely unknown. Thanks to TCGA, large scale analyses of differences between male and female patients become possible and start to emerge [22–25]. For a recent review on sexual dimorphism in cancer, see [26]. Knowing when features that distinguish tumor types differ between genders might enhance the utility of such features as biomarkers.

We undertook a comprehensive pan-cancer classification of 9096 tumor samples from 31 tumor types from TCGA using RNA-seq gene expression data. We aimed to identify a set of genes whose expression levels can classify all 31 TCGA pan-cancer tumor types. We also carried out the same pan-cancer classification on the gene expression data from 602 “normal” tissue samples taken adjacent to tumors for 17 tumor types. We compared the top-ranked discriminative genes from both tumor and “normal” samples and concluded that most discriminative genes that we identified reflected tumor-type differences rather than simply tissue-of-origin differences. Moreover, we sought to identify, separately in men and in women, analogous sets of genes that can distinguish the 23 sex non-specific tumor types. We hope to gain insight into sexual dimorphism in some tumors from those analyses.

## Data

We downloaded all (March, 2015) UNC RNASeqV2 level 3 expression data from the TCGA data portal (https://tcga-data.nci.nih.gov) for 9096 patients representing 31 tumor types (Table 1) and for 602 “normal” samples taken adjacent to tumors representing 17 tumor types (Additional file 1: Table S1). We log2-transformed the TCGA normalized read counts (rsem.genes.normalized) for the RNA-seq data (Because read depths ≤1 Reads Per Kilobase per Million are mostly noise, we filtered them by assigning all values less than 1 the value 1 before transformation.).

**Table 1:** Tumor types and number of TCGA RNA-seq samples used in the analysis

Available Cancer Types		Number of Samples		
		Pan-cancer	Males (%)	Females (%)
Adrenocortical carcinoma	ACC	79	31 (0.76)	48 (1.82)
Bladder urothelial carcinoma	BLCA	408	272 (6.67)	99
Breast invasive carcinoma	BRCA	1102	Sex-specific (omitted)	
Cervical squamous cell carcinoma and endocervical adenocarcinoma	CESC	306	Sex-specific (omitted)	
Cholangiocarcinoma	CHOL	36	Too few (omitted)	
Colon adenocarcinoma	COAD	287	156 (3.82)	129 (4.89)
Lymphoid neoplasm diffuse large B-cell lymphoma	DLBC	48	Too few (omitted)	
Esophageal carcinoma	ESCA	Not available	159 (3.90)	26 (0.99)
Glioblastoma multiforme	GBM	169	109 (2.67)	59 (2.24)
Head and Neck squamous cell carcinoma	HNSC	522	385 (9.43)	137 (5.19)
Kidney chromophobe	KICH	66	Too few (omitted)	
Kidney renal clear cell carcinoma	KIRC	534	346 (8.48)	188 (7.13)
Kidney renal papillary cell carcinoma	KIRP	291	214 (5.24)	77 (2.92)
Acute Myeloid Leukemia	LAML	173	93 (2.28)	80 (3.03)
Brain lower grade glioma	LGG	534	292 (7.16)	241 (9.14)
Liver hepatocellular carcinoma	LIHC	374	253 (6.20)	121 (4.59)
Lung adenocarcinoma	LUAD	517	240 (5.88)	277 (10.50)
Lung squamous cell carcinoma	LUSC	502	371 (9.09)	131 (4.97)
Mesothelioma	MESO	87	71 (1.74)	16 (0.61)
Ovarian serous cystadenocarcinoma	OV	266	Sex-specific (omitted)	
Pancreatic adenocarcinoma	PAAD	179	99 (2.43)	80 (3.03)
Pheochromocytoma and Paraganglioma	PCPG	184	82 (2.01)	102 (3.87)
Prostate adenocarcinoma	PRAD	498	Sex-specific (omitted)	
Rectum adenocarcinoma	READ	95	52 (1.27)	42 (1.59)
Sarcoma	SARC	263	119 (2.92)	144 (5.46)
Skin cutaneous melanoma	SKCM	473	259 (6.35)	156 (5.91)
Stomach adenocarcinoma	STAD	Not available	268 (6.57)	147 (5.57)
Testicular germ cell tumors	TGCT	156	Sex-specific (omitted)	
Thyroid carcinoma	THCA	513	102 (2.50)	246 (9.33)
Thymoma	THYM	120	63 (1.54)	57 (2.16)
Uterine corpus endometrial carcinoma	UCEC	177	Sex-specific (omitted)	
Uterine carcinosarcoma	UCS	57	Too few (omitted)	
Uveal melanoma	UVM	80	45 (1.10)	35 (1.33)
Total		9096	4081	2638

For the sex non-specific tumor classification, we eliminated all tumor types that are sex-specific, namely, BRCA, CESC, OV, PRAD, TGCT, UCEC and UCS. For the remaining tumor types, we separated samples into two groups based on the patients’ gender. We then eliminated three additional tumor types (CHOL, DLBC and KICH) due to small gender-specific sample sizes. At the time of analyses, data for two new tumor types (ESCA and STAD) became available and were included in the analysis. This brought the total number of sex non-specific tumor types to 23 with 2638 females and 4081 males RNA-seq samples. The numbers of samples for each tumor type from each gender are listed in Table 1.

## Methods

We used the GA/KNN method [27, 28] for pan-cancer classification. GA/KNN employs a genetic algorithm (GA) as the gene/feature selection engine and the *k*-nearest neighbors (KNN) algorithm as the classification tool. GA/KNN can identify gene signatures that not only can separate different classes of samples but also may uncover subtypes within a class. One valuable characteristic of GA/KNN is that, for each training/testing partition, it identifies many near-optimal feature sets and uses each feature set to predict the testing-set samples. Because the algorithm classifies each sample multiple times, one can calculate the proportion of times that each sample was predicted to be each of the 31 classes plus a category of unclassifiable due to ties (proportions sum to 1). Furthermore, one can also assess the relative importance of each gene for sample classification by counting how often that gene appears in those near-optimal feature sets.

In a genetic algorithm, the “chromosome” encodes the candidate solution - the gene signature in this case. A collection of “chromosomes” is referred to as a “population”. In this analysis, the chromosome length was set to 20 (a 20-gene set). The population size was set to 300 chromosomes. The maximal number of “generations” was set to be 300. For KNN classification, *k* was set to 5 with a majority “voting” rule. We selected these parameters based on an earlier comprehensive analysis of the effect of the choice of parameters on both gene selection and classification accuracy [27].

We randomly divided the data into a training (75% of the samples, e.g., ~6800 samples for pan-cancer classification) and a testing set (25% of the samples, ~2300 samples) with samples drawn proportionally from each tumor type without replacement. The genetic algorithm stopped either when the best “chromosome” in the current “population” classified at least 90% of the training samples correctly or when the search reached a predefined maximal number of “generations” (see below). We refer to the resulting gene set as a near-optimal classifier. The near-optimal classifier was subsequently used to predict the class membership of the samples in the testing set. The predicted and actual class memberships were then compared to calculate the testing-set prediction accuracy. Because the number of features (genes) is much larger than the number of samples (commonly referred to as small *n* large *p*), multiple equally discriminative feature sets may exist. We repeated the above GA/KNN procedure 1000 times with the training and testing partitioning unchanged, resulting in 1000 near-optimal classifiers (not necessarily distinct) and 1000 testing prediction accuracies.

The prediction accuracy may vary depending on which samples are assigned to the training set. Given the large size of the pan-cancer gene expression dataset and the high computational demand of the algorithm, we only repeated the above procedure twice, each with an independent training/testing partition to avoid idiosyncrasies from use of a single random assignment. For the sex non-specific pan-cancer classification, we were able to repeat the above procedures five times each for males and for females because of the sample size reduction. For each gender, we combined results from all five independent training/testing partitions. Specifically, if a sample appeared in more than one test set, we averaged the results (see below).

To assess whether the top-ranked discriminative genes that we identified from the tumor samples were specific to the tumors themselves or to the tissue type where the tumors originated, we carried out the same “pan-cancer” classification on the gene expression data from 602 “normal” RNA-seq samples representing 17 tissue types (Additional file 1: Table S1). In addition, we used these “normal” samples to compare performance between GA/KNN and a gradient boosting-based classifier named XGBoost [29]. Specifically, we randomly generated 10 different training/testing partitions with 75% of samples as training and 25% as testing; samples were draw proportionally to their class size.

For our GA/KNN analysis of the “normal” samples, we used the same parameter settings as for the tumor samples. To decide on parameter settings for XGBoost, we first carried out a grid search for the optimal hyperparameters over ranges that we believed were close to optimal from our previous experience with XGBoost on gene expression data. We used 10-fold cross-validation (repeated 10 times) on all RNA-seq samples and chose as optimal the hyperparameters that gave the best averaged cross-validation results (Additional file 2: Table S2). For our XGBoost analyses, we set the number of trees (boosts) to 200, with early stopping criteria when the minimum training error did not improve in 20 rounds. The average number of boosts needed was ~19 (minimum = 7 and maximum = 46). Since XGBoost is a stochastic classifier, we ran XGBoost with the optimal hyperparameters for 1000 times for each of the 10 training/testing partitions. We rank all genes based on the average of times a gene is selected to build the forest from all repeated runs. For each of the 10 testing datasets, we computed the classification accuracy.

All results presented in the remainder of the manuscript are based on samples from testing sets that were not involved in the training process.

## Results

### Pan-Cancer classification of all tumors ignoring gender

A sample may be unclassifiable by KNN due to the failure of any single tumor type to be in the majority among its nearest neighbors. Thus, given a test sample, it could be classified into one of the 31 tumor types or this unclassifiable category. When the GA/KNN algorithm is applied in many independent runs (here 2000), the proportion of times each sample is predicted to be each of the 32 classes can be obtained (Additional file 3: Table S3). Those 32 proportions sum to 1. One among them is the proportion of GA/KNN runs that a sample was predicted to be its own type, i.e., correctly classified (bolded in Additional file 3: Table S3). For simplicity, we referred to this proportion as proportion-times-correctly-classified (denoted π_cc_) throughout the manuscript. Summary statistics for π_cc_ for each tumor type are shown in Table 2.

**Table 2:** Summary statistics for π_cc_ values when classifying 31 tumor types and ignoring sex of the samples across 1000 GA/KNN runs for each of two training/testing partitions (2000 runs total)

Type	Minimum	1st Quartile	Median	Mean	3rd Quartile	Maximum	Modal Prediction Accuracy
ACC	0.23	0.76	0.88	0.83	0.92	0.97	0.97
BLCA	0.01	0.51	0.81	0.71	0.96	1.00	0.91
CHOL	0.00	0.01	0.40	0.37	0.50	0.66	0.73
COAD	0.18	0.77	0.85	0.83	0.91	0.98	0.99
DLBC	0.65	0.82	0.89	0.87	0.94	0.98	1.00
GBM	0.46	0.86	0.96	0.91	0.98	1.00	0.99
HNSC	0.04	0.91	0.98	0.93	1.00	1.00	0.99
KICH	0.00	0.88	0.92	0.86	0.96	0.99	0.96
KIRC	0.00	0.98	1.00	0.93	1.00	1.00	0.96
KIRP	0.00	0.79	0.97	0.85	1.00	1.00	0.92
LAML	0.89	1.00	1.00	0.99	1.00	1.00	1.00
LGG	0.56	0.99	1.00	0.97	1.00	1.00	1.00
LHIC	0.04	0.97	0.99	0.94	1.00	1.00	0.98
LUAD	0.00	0.88	0.96	0.88	0.99	1.00	0.96
LUSC	0.03	0.67	0.92	0.78	0.97	1.00	0.88
MESO	0.00	0.72	0.87	0.76	0.93	1.00	0.90
PAAD	0.03	0.84	0.96	0.85	0.99	1.00	0.95
PCPG	0.71	0.98	1.00	0.98	1.00	1.00	1.00
READ	0.03	0.09	0.14	0.15	0.19	0.28	0.00
SARC	0.03	0.78	0.91	0.83	0.96	1.00	0.96
SKCM	0.00	0.93	0.97	0.90	0.99	1.00	0.97
THCA	0.37	1.00	1.00	0.99	1.00	1.00	1.00
THYM	0.08	0.90	0.99	0.89	1.00	1.00	0.94
UCS	0.01	0.06	0.26	0.27	0.41	0.62	0.62
UVM	0.52	0.95	0.99	0.95	1.00	1.000	1.00
BRCA	0.01	0.98	0.99	0.97	1.00	1.00	0.99
CESC	0.00	0.52	0.76	0.68	0.87	0.98	0.94
OV	0.36	0.95	0.98	0.95	0.99	1.00	1.00
PRAD	0.53	1.00	1.00	0.99	1.00	1.00	1.00
TGCT	0.25	0.97	1.00	0.94	1.00	1.00	1.00
UCEC	0.04	0.52	0.71	0.68	0.86	1.00	0.96

The rightmost column labeled “Modal Prediction Accuracy” is not based on π_cc_ but instead on a prediction using the tumor type to which each sample was assigned most often

**Fig. 1:**
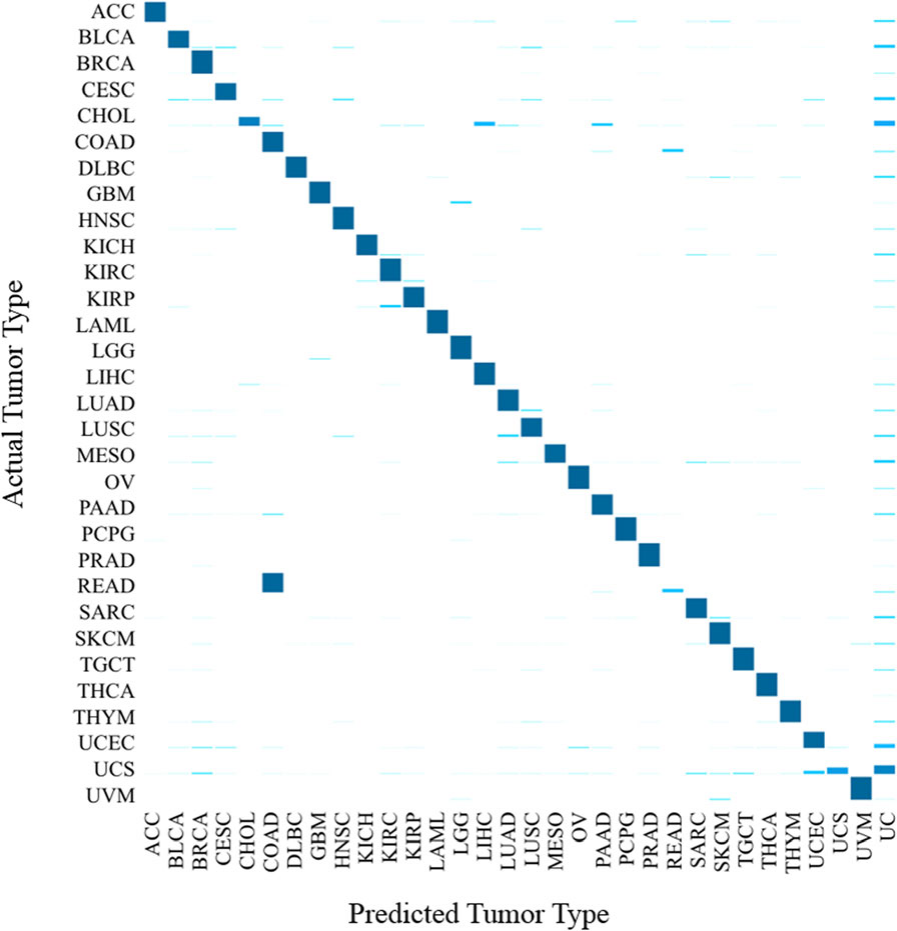
Proportion of test-set samples predicted to be each of the 31 tumor types. Y-axis lists the 31 actual tumor types; x-axis lists the 32 possible classification categories (31 tumor types plus “unclassified” [UC]). Each *bar* represents one of the 32 proportions that samples from the actual tumor type were predicted to be. The 32 plotted proportions represent means from the corresponding proportions for all samples of the actual tumor type

The median value of π_cc_ across samples from a given tumor type was in the range of 90–100% for most tumor types. Tumor types such as DLBC, BRCA, LAML, LGG, PCPG, OV, THCA, and UVM had among the highest median π_cc_ values, suggesting that those tumor types could be easily distinguished from all others. For example, BRCA samples were overwhelmingly correctly predicted to be BRCA (Fig. 1). In contrast, the median π_cc_ values for CHOL, READ and UCS were rather low (0.400, 0.136, and 0.255), indicating that those tumors were often classified to types other than themselves (Fig. 1). A close examination showed that the reasons for the low proportions among the four tumor types were not the same. For CHOL, the π_cc_ were the largest among the 32 proportions for 11 of the 15 test samples, suggesting that those samples were still likely to be assigned to CHOL. Among the four misclassified samples, one (TGCA-W5-AA39) was consistently mis-assigned to LIHC (liver) and one (TCGA-3X-AAV9) to PAAD (pancreatic). No clear patterns were seen for the remaining two. For READ, all samples were most often mis-assigned to COAD. About half of the UCS samples were mis-assigned, most often to “unclassifiable” or to UCEC. Samples from three kidney tumor types (KICH, KIRC, and KIRP) were largely correctly classified; those misclassified were assigned to the other kidney tumor types, rather than to tumors in different organs. This mis-assignment within organ was also true for the two lung tumor types (LUAD and LUSC). In essence, the main cause for misclassification among the tumor types appeared to be similarity in their tissue of origin.

The above analysis was explicitly based on the π_cc_ values. As an alternative way to assess accuracy, we can proceed as follows. For each test-set sample, we can determine its predicted tumor type for each of the 2000 GA/KNN runs and use that information to determine the modal prediction, the tumor type to which the sample was assigned most often. If the modal prediction matched the actual tumor type, we regarded the prediction as correct. The proportion of correct predictions across all the samples of a given tumor type measures what we call the modal prediction accuracy for that tumor type. These modal prediction accuracies (rightmost column in Table 2) are often higher than the corresponding median value of π_cc_. Averaging across all tumor types, the overall modal prediction accuracy was 95.6% (weighted by number of samples in each tumor type).

### Top-ranked genes

**Fig. 2:**
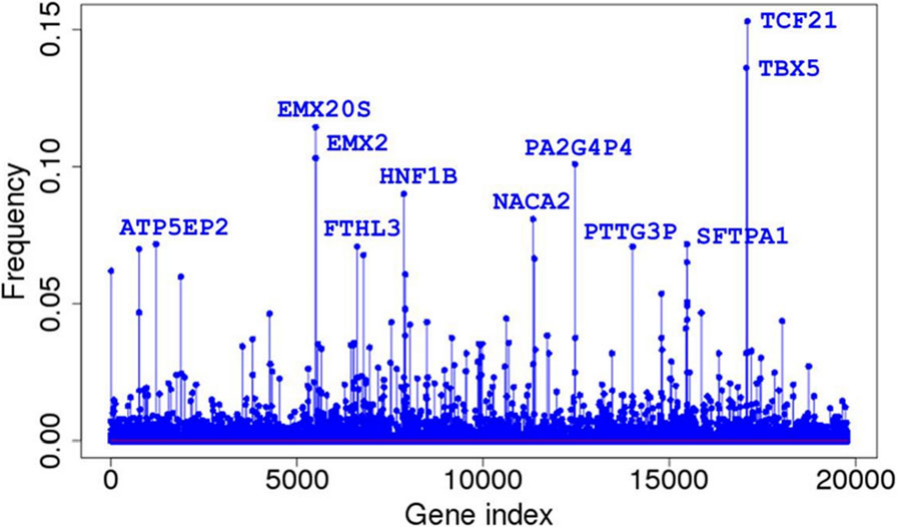
Stem plot of gene selection frequency based on 2000 near optimal gene selection classifiers from 1000 GA/KNN runs for each of two training/testing partitions

From each of the two independent training/testing partitions, we obtained 1000 sets (a set consists of 20 genes) of near-optimal classifiers (2000 sets altogether); and we calculated the frequency with which each gene appeared in those sets (Fig. 2). We regard the frequencies as indicative of the importance of the corresponding genes for sample classification. Remarkably, there are 40 genes in the intersection of top 50s from both partitions and 60 genes in the union of top 50s, indicating that our results were largely reproducible. Those genes from one partition that did not appear in the top 50 from the other partition were all among the top 100 in the other partition, variation likely attributable to the stochastic nature of the algorithm. We combined the counts from the two independent runs. Gene ontology analysis of the top 200 genes in the combined list suggested that those genes are highly enriched in genes implicated in the biological process of development (Table 3).

**Table 3:** Enriched gene ontology (GO) terms for the top 200 genes from the pan-cancer classification of all 9096 samples ignoring the gender

Gene ontology (GO) terms	*P*-value
Anatomical structure development	3.2e-10
Anatomical structure morphogenesis	3.7e-10
Developmental process	5.0e-10
System development	1.1e-9
Tissue development	1.4e-9
Organ development	2.7e-9
Multicellular organismal development	3.7e-9
Epithelium development	2.5e-7
Tube development	1.2e-6
Regulation of transcription, DNA-dependent	1.5e-6

**Fig. 3:**
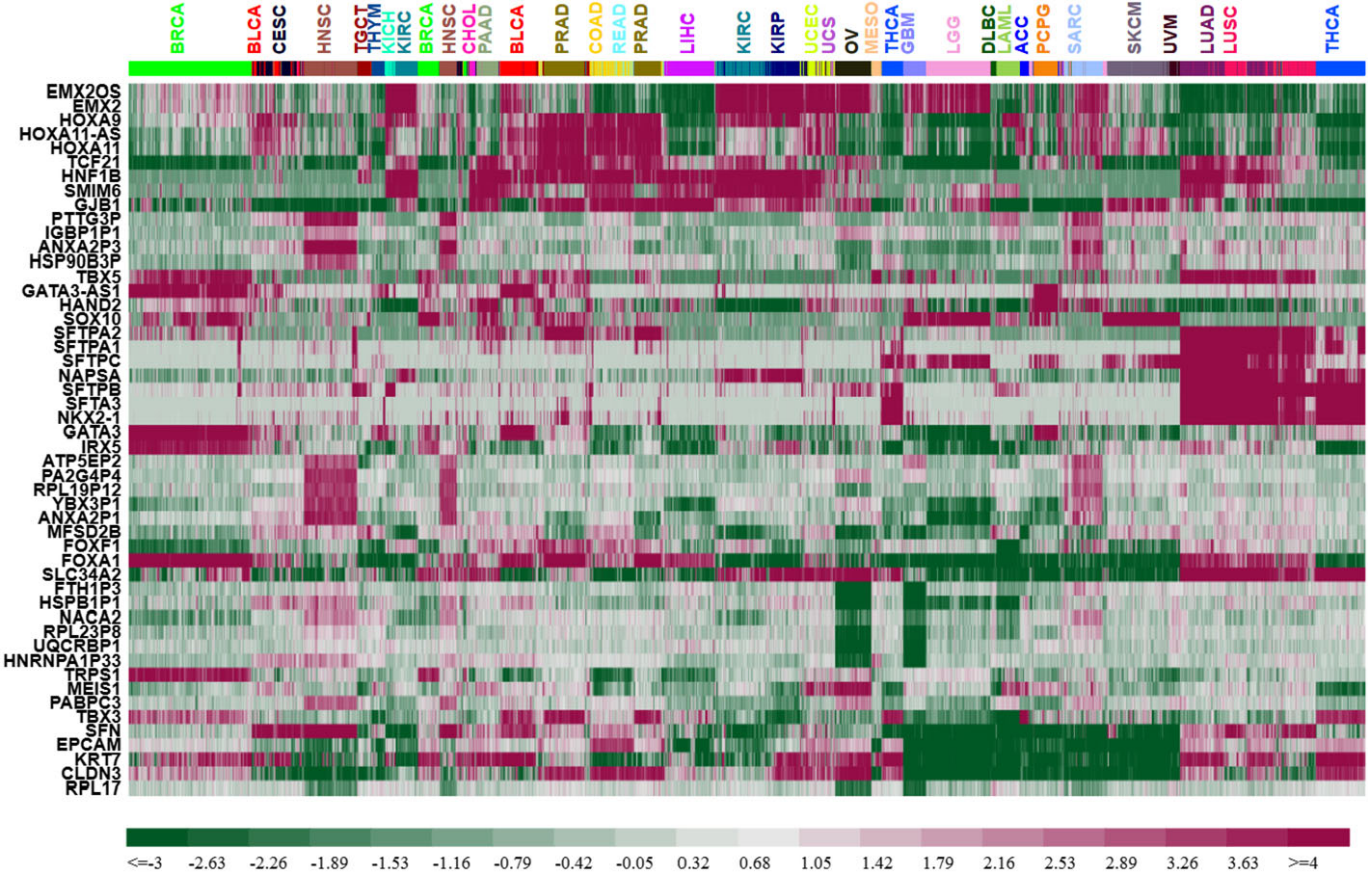
Heatmap representation of the expression patterns of the top 50 genes across all 9096 samples. Each row (gene) was centered by the median expression value across all samples. A hierarchical clustering analysis was carried out for both samples and genes using the Euclidean distance as the similarity metric

The 20 most frequently selected genes were *TCF21*, *TBX5*, *EMX20S*, *EMX2*, *PA2G4P4*, *HNF1B*, *ATP5EP2*, *NACA2*, *PTTG3P*, *FTH1P3*, *SFTPA1*, *HSPB1P1*, *GATA3*, *NAPSA*, *ANXA2P3*, *IGPB1P1*, *HOXA9*, *STFA3*, *RPL19P12*, and *SFTPA2*. A heatmap representation of the relative expression levels of the top 50 genes across all 9096 tumor samples is shown in Fig. 3.

*TCF21*, the most frequently selected gene, encodes a transcription factor of the basic helix-loop-helix family. The TCF21 product is mesoderm specific and expressed in embryonic epicardium, mesenchyme-derived tissues of lung, gut, gonad, and both mesenchymal and glomerular epithelial cells in the kidney. It is required for normal heart development [30–32]. *TBX5*, a member of the T-box genes, encodes a transcription factor that is involved in the regulation of developmental processes.

Five surfactant genes (*SFTA3*, *SFTPA1*/*A2*, and *SFTPB*/*C*) were among the top 50. All five genes were highly expressed in LUAD and LUSC and low in all other tumors except that *SFTPB* and *STFA3* were also highly expressed in THCA. Very few other genes showed such tumor specificity.

About one third of the top 50 genes encode transcription factors (TFs) and another one third encode proteins involved in cell adhesion, ion and small molecular transport, protein synthesis and folding, and lung function. Surprisingly, the final third contains 14 pseudogenes and two antisense non-coding genes (*EMX2OS* and *GATA3-AS1*).

### Pseudogenes

Pseudogenes were significantly enriched among the top 50 genes (14 pseudogenes) (*P* = 1.2 × 10^−19^, hypergeometric test) as well as among the top 100 genes (27 pseudogenes) (*P* = 1.5 × 10^−17^).

The top-ranked pseudogene was *PA2G4P4* (proliferation-associated 2G4 pseudogene 4). Its functional counterpart is *PA2G4* (proliferation-associated 2G4). PA2G4 is an RNA-binding protein present in pre-ribosomal ribonucleoprotein complexes and is involved in growth regulation. PA2G4P4 was highly expressed in nearly all tumor samples with the overall highest expression in HNSC, OV, and SARC. Expression levels of *PA2G4P4* were positively correlated with those of *PA2G4* in all tumor types except KIRC, PRAD, and THCA (Additional file 4: Figure S1) – remarkable given that *PA2G4* is on chromosome 12 and *PA2G4P4* is on chromosome 3. The expression of *PA2G4P4* was also correlated with that of *NACA2* in about one third of the tumor types (data not shown). *NACA2*, the nascent polypeptide associated complex alpha subunit 2 gene, is, like *PA2G4*, involved in growth regulation and RNA processing.

Interestingly, none of the functional counterparts of the 14 pseudogenes was among the top 100 most frequently selected genes, indicating that the expression of the pseudogenes might better discriminate tumor types than expression of their functional counterparts.

### Putative tumor subtypes

Tumors are heterogeneous [33]. For example, breast cancer is known to have several distinct subtypes [12–14, 34]. To see if the top-ranked genes for pan-cancer classification can further uncover tumor subtypes, for each tumor type we carried out *k*-means clustering using expression data for the 50 top-ranked pan-cancer genes that had non-zero interquartile ranges for the particular tumor type. Consequently, each tumor type had a slightly different 50-gene set. In this exploratory analysis, we set the *k* = 2 or *k* = 3, that is, we allowed two or three subgroups for each tumor type. We selected an optimal *k* using the silhouette method [35]. All tumor types can be divided into 3 sub-groups based on the minimum averaged silhouette scores, except SARC and THYM. In addition, we have excluded tumor types GBM, LAML, and OV that have incomplete survival data. Based on the survival analyses, the tumors which have putative subgroups that are correlated with survival after the Bonferroni correction (i.e., *P* < 0.001) (Additional file 5: Methods) included ACC, BLCA, BRCA, KIRC, KIRP, LGG, and PAAD.

The heatmaps of ACC, BLCA, BRCA, KIRC, KIRP, LGG, and PAAD from *k*-mean clustering analysis are shown in Additional file 6: Figure S2 and the subgroups associations to survival outcomes are highlighted in Additional file 7: Figure S3. BRCA tumors have distinct subtypes, and the subtypes are associated with survival outcomes [13]. Using the top-ranked genes, we largely recapitulated the ER+ and basal-like subtypes (Additional file 6: Figure S2c) although the association between patients’ survival and our subtypes is only marginally significant after multiple testing adjustment (*P* = 0.008, Additional file 7: Figure S3c). It is worth pointing out that the subset of genes used in subtype discovery was identified from pan-cancer classification only.

### Classification of samples of “normal” tissue taken adjacent to tumors

Using GA/KNN, we correctly classified on average 87.6% test set “normal” samples. The heatmap representation of the relative expression levels of the top 50 genes across all 602 “normal” samples are shown in Additional file 8: Figure S4. Among the 100 top-ranked discriminative genes for “normal” samples using GA/KNN, only 18 were in common with the top 100 discriminative genes from tumor samples (*C11orf9*, *EMX2*, *EMX20S*, *ESR1*, *FOXF1*, *FTHL3*, *GAL3ST1*, *HAND2*, *HOXA11*, *HOXA11AS*, *HOXA9*, *IRX5*, *NACA2*, *NBLA00301*, *PA2G4P4*, *SFTPD*, *TBX5*, and *TCF21*). Restricting to the top 50 discriminative genes, the corresponding overlap was eight. This result suggests that most genes that we identified as distinguishing among the 31 tumor types are differences among the tumor types themselves and not simply reflecting differences among the tissues where the tumors originated.

Comparisons of classification accuracy for GA/KNN and XGBoost for 10 testing sets are shown in Additional file 9: Figure S5. The averaged accuracies are comparable between the two methods (87.6% for GA/KNN vs 90.2% for XGBoost). The heatmap representation of the relative expression levels of the top 50 genes selected by XGBoost across all 602 “normal” samples is shown in Additional file 10: Figure S6. Among the 100 top-ranked discriminative genes from GA/KNN on “normal” samples and corresponding top 100 -from XGBoost, only 16 were in common (*C15orf21*, *C19orf20*, *CALML3*, *DSG3*, *FOXF1*, *FOXL2*, *FTHL3*, *KIF12*, *KRT6A*, *NACA2*, *PCSK1N*, *SCARNA9*, *SFTPD*, *TCF23*, *TSSK6*, and *ZMYND17*).

### Pan-Cancer classification of sex non-specific tumors

For the 23 sex non-specific tumor types, we had 4081 samples from males and 2638 from females. The sample imbalance favoring males persisted in most individual tumor types except for ACC, LUAD, PCPG, SARC, and THCA (Table 1). For each gender, we carried out 1000 independent GA/KNN runs for each of five independent training/testing partitions.

The quartiles for the π_cc_ values for males and females over the 5000 total runs are listed in Table 4 (Additional file 11: Figure S7). Overall, the results recapitulated those from our pan-cancer analysis of 31 tumor types that ignored gender. Those tumor types with high prediction accuracy remained high and those with low accuracy stayed low regardless whether gender was considered or ignored. All READ samples were predicted to be COAD for both genders. The prediction accuracies for BLCA, ESCA, and MESO were relatively low compared to other tumor types regardless of gender.

**Table 4:** Quartiles for π_cc_ values when classifying 23 non-sex-specific tumor types separately using male and female samples across 1000 GA/KNN runs for each of five training/testing partitions (5000 runs total)

Type	Minimum		1st Quartile		3rd Quartile		Maximum		Modal Prediction Accuracy	
	F	M	F	M	F	M	F	M	F	M
ACC	0.01	0.01	0.83	0.61	0.95	0.84	1.00	0.95	0.97	0.93
BLCA	0.01	0.04	0.46	0.66	0.91	0.96	1.00	1.00	0.89	0.93
COAD	0.22	0.12	0.83	0.83	0.93	0.91	1.00	0.96	1.00	0.99
GBM	0.35	0.59	0.91	0.96	0.99	1.00	1.00	1.00	1.00	1.00
HNSC	0.54	0.06	0.93	0.96	1.00	1.00	1.00	1.00	1.00	1.00
KIRC	0.00	0.00	0.98	0.98	1.00	1.00	1.00	1.00	0.95	0.98
KIRP	0.00	0.00	0.51	0.93	0.92	1.00	1.00	1.00	0.89	0.92
LAML	0.93	0.83	1.00	0.99	1.00	1.00	1.00	1.00	1.00	1.00
LGG	0.72	0.16	0.99	0.99	1.00	1.00	1.00	1.00	1.00	1.00
LIHC	0.01	0.01	0.93	0.98	1.00	1.00	1.00	1.00	0.99	0.99
LUAD	0.06	0.05	0.90	0.80	0.99	0.96	1.00	1.00	0.96	0.97
LUSC	0.00	0.03	0.49	0.85	0.94	0.99	1.00	1.00	0.86	0.94
MESO	0.03	0.00	0.50	0.78	0.79	0.95	0.89	1.00	0.92	0.95
PAAD	0.00	0.08	0.84	0.83	0.99	0.99	1.00	1.00	0.95|	0.93
PCPG	0.14	0.80	0.98	0.97	1.00	1.00	1.00	1.00	0.98	1.00
READ	0.01	0.03	0.08	0.09	0.15	0.17	0.33	0.23	0.00	0.00
SARC	0.17	0.06	0.88	0.83	0.98	0.95	1.00	1.00	1.00	0.99
SKCM	0.15	0.01	0.89	0.90	0.98	0.98	1.00	1.00	0.98	0.97
THCA	0.81	0.71	1.00	0.96	1.00	1.00	1.00	1.00	1.00	1.00
THYM	0.09	0.08	0.85	0.89	1.00	0.99	1.00	1.00	0.98	0.92
UVM	0.76	0.28	0.95	0.92	0.99	0.99	1.00	1.00	1.00	0.97
ESCA	0.00	0.05	0.02	0.35	0.61	0.97	0.80	1.00	0.38	0.64
STAD	0.07	0.08	0.84	0.80	0.98	0.96	1.00	1.00	0.98	0.97

The rightmost column labeled “overall” is not based on π_cc_ but instead on a prediction using the tumor type to which each sample was assigned most often

To see the subtle differences between males and females, here we considered the top 100 genes from each gender. The union for the top 100 genes from males and from females contained 125 genes and the intersection contained 75 genes (Additional file 12: Table S4). Two heatmap representations of the relative expression levels of the top genes across all male and female tumor samples are shown in Additional file 13: Figure S8. Many genes had similar ranks in both genders; 21 differed by more than 100 in rank (Table 5). Rank sum tests showed that all 21 genes were differentially expressed in samples between males and females in at least one tumor type (data not shown). In the following paragraphs, we focus on genes that were largely differentially expressed between females and males in tumor samples and whose possible role in sexual dimorphism received support in existing literature.

**Table 5:** Gene ranks from full female dataset, full male dataset, and the eight “matched” male datasets

	Gene	Rank from full female dataset	Rank from full male dataset	Difference (F-M)	Mean (SD) rank from 8 matched male datasets	Difference (F-meanM)
Genes ranked higher using male samples than female samples	BNC1	932	45	887	54 (16)	878
	FAT2	392	90	302	143 (23)	249
	KRT5	328	47	281	165 (57)	163
	RNF43	299	94	205	81 (14)	218
	S1PR5	281	99	181	98 (38)	183
	ANKS4B	245	96	148	115 (20)	130
	CSTA	218	93	125	129 (33)	89
	ANXA8	161	48	113	121 (36)	40
	KRT8	175	65	110	94 (22)	81
	CLRN3	204	98	106	86 (15)	118
Genes ranked higher using female samples than male samples	FOXA1	82	417	−335	237 (92)	−155
	AMY1A	100	370	−270	386 (162)	−286
	HPN	74	336	−262	256 (94)	−182
	LAD1	45	269	−224	129 (40)	−84
	PDZK1	83	293	−210	228 (79)	−145
	TMC5	55	241	−186	139 (50)	−84
	KIF12	89	249	−160	324 (135)	−235
	STK32A	79	226	−147	123 (28)	−44
	CFAP221	81	187	−106	94 (21)	−13
	TRIM29	86	188	−102	143 (25)	−57
	HOXA11	84	184	−100	291 (77)	−207

**Fig. 4:**
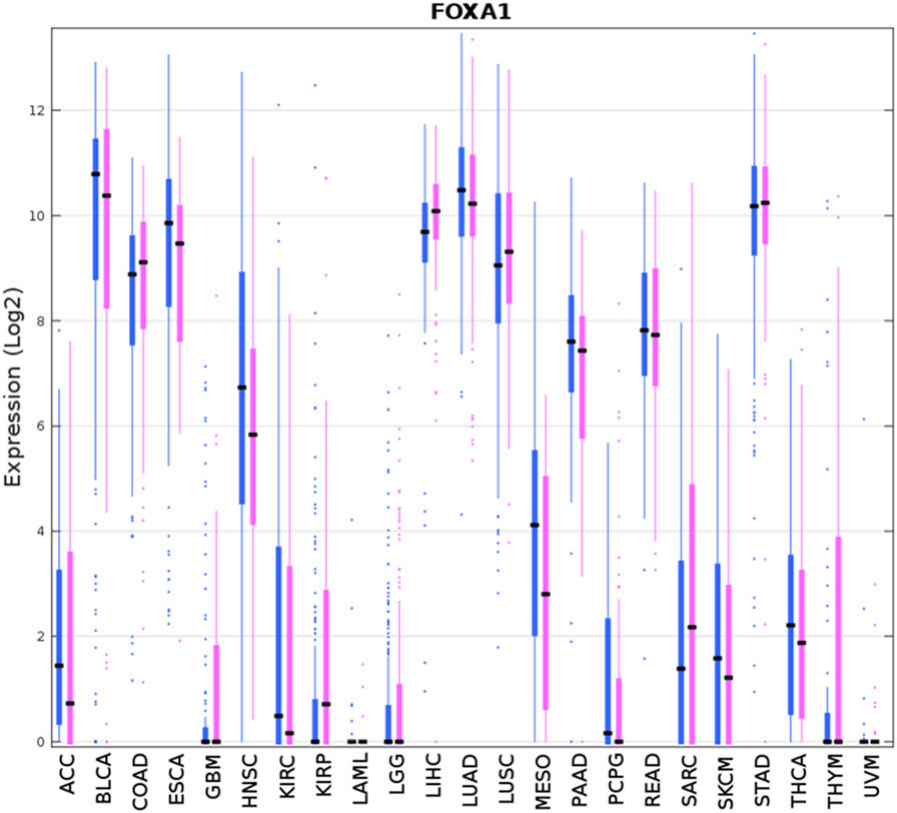
Boxplots of *FOXA1* expression data in the 23 sex non-specific tumors from males (*blue*) and females (*pink*)

*FOXA1* had rank 82 in females and 417 in males, suggesting that *FOXA1* expression level might be more important for distinguishing sex non-specific tumors in females than in males. *FOXA1* had significantly higher expression in LIHC in females than in males (*P* = 9.9 × 10^−5^, rank sum test, two-sided). Li et al. [22] elegantly showed that *FOXA1*/*A2* transcription factors regulate estrogen signaling differently in liver and mammary gland, that this female hormone is protective for liver cancer in mice and that this protection requires *FOXA1*/*A2*. Upon exposure to hepatocarcinogens, the tumor load in mutant *FOXA1*/*A2* female mice was dramatically increased whereas the tumor load in mutant *FOXA1*/*A2* male mice was dramatically decreased [22]. Besides LIHC, *FOXA1* had significantly higher expression in HNSC (*P* = 2.4 × 10^−3^, rank sum test, two-sided) and KIRP (*P* = 6.5 × 10^−4^, rank sum test, two-sided) in females than in males (Fig. 4). Whether *FOXA1* might also play a role for sexual dimorphism in HNSC and KIRP remains unclear.

On the other hand, *BNC1* ranked high in males (45th) and low in females (932nd). BNC1 (basonuclin 1) is a zinc finger protein that is thought to play a regulatory role in epithelial proliferation. BNC1 modulates TGF-β1-induced epithelial dedifferentiation of mammary epithelial cells [36]. *BNC1* had significantly higher expression in females than in males (Additional file 14: Figure S9) in HNSC (*P* = 8.7 × 10^−3^, rank sum test, two-sided), LIHC (*P* = 5.4 × 10^−5^, rank sum test, two-sided), and THCA (*P* = 2.4 × 10^−3^, rank sum test, two-sided). Interestingly, *BNC1* is also a putative ER receptor 1 (ESR1) target as ESR1 was bound in the proximal promoter of *BNC1* in T-47D cell line (ENCODE data on UCSC genome browser), raising the possibility that BNC1 might also play a role in sexual dimorphism in liver cancer similar to FOXA1.

Among the 21 genes, *FAT2* had larger differential expression between males and females in KIRP (*P* = 7.5 × 10^−7^, rank sum test, two-sided) than in any other sex non-specific tumor. *FAT2* encodes a tumor suppressor essential for controlling cell proliferation during Drosophila development [37]. FAT2 is a member of the cadherin superfamily and most likely functions as a cell adhesion molecule [38]. *FAT2* was frequently mutated in clear cell renal cell carcinoma [39, 40]. It is not clear whether FAT2 plays a role in sexual dimorphism in KIRP.

To see if the differences that we observed in both prediction accuracy and gene ranks between males and females were due to the imbalance of sample proportions among the tumor types, we generated eight male datasets that matched approximately both the total number of samples and tumor proportions as those in females by taking random samples from males without replacement. We repeated the same pan-cancer classification procedure on each of the eight “matched” male datasets as above. The mean and median π_cc_ values from the full female dataset, the full male dataset and the eight “matched” male datasets are shown in Additional file 15: Table S5. Female-male differences in mean or median π_cc_ values observed from original dataset were strongly reduced (Additional file 15: Table S5) when the sample proportions were balanced between the genders. The difference in gene ranks remained (Table 5), however. Basically, genes, such as *BNC1* that ranked high from the full male dataset remained high from the matched male datasets, and those ranked low remained low, although a shrinking of rank differences is also apparent (Table 5).

## Discussion

Gene expression data can be used to classify tumor types and uncover tumor subtypes that may suggest targeted treatment options. We carried out a pan-cancer classification of ~9100 TCGA tumors from 31 tumor types using RNA-seq gene expression data. We found that, among the 31 tumor types, BRCA, GBM, HNSC, KIRC, LAML, LGG, LIHC, OV, PCPG, PRAD, SKCM, THCT, THCA, THYM, and UVM were more easily distinguished from all other tumor types. Tumors from similar tissue origins (e.g., READ and STAD; UCS and UCEC) are usually more difficult to distinguish from each other than those from different lineages (e.g., READ vs LAML). In an extreme, nearly all READ samples were indistinguishable from COAD samples. Surprisingly, the three kidney tumors (KICH, KIRC, and KIRP) were distinguished from each other and from all other tumor types using gene expression data alone.

We were able to correctly classify more than 90% of the tumor samples overall using many different 20-gene sets, though some genes appeared repeatedly in the sets. Both sample prediction accuracies and gene rank (a measure of importance in classification) were largely reproducible.

We showed that the top ranked genes from the pan-cancer analysis were able not only to distinguish different types of tumor samples but also to uncover potential subtypes within some tumor types. The top 50 genes from our analysis largely captured the ER positive luminal A or luminal B and ER negative basal-like subgroups – subgroups that have distinct survival profiles. For BLCA, KIRC, KIRP, LGG, and PAAD, patients in the three putative subgroups had differential survival.

Our primary analysis tool was GA/KNN, a supervised classification method that carries out feature selection and classification simultaneously [27, 28]. To compare its performance with a more recent supervised method, XGBoost (gradient boosting machines), we ran both tools on ten training/testing partitions generated from the 602 “normal” RNA-seq samples. Test set classification accuracy (~90%) was comparable for both methods. Despite similar classification performance, the top-ranked discriminative genes derived from two methods showed little overlap. Clearly multiple sets of genes may give similar classification performance, but which tool provides a gene list with more biological relevance or utility beyond classification remains an open question – and one that is impossible to address by algorithmic methods alone.

Though unlikely by chance, one third of the top 50 genes were pseudogenes. Interestingly, none of their functional counterparts ranked among the top 100 genes, suggesting that those pseudogenes may better serve as features than their functional counterparts in distinguishing among tumor types. Pseudogenes share high sequence homology to their functional counterparts but in most cases contain deletions/insertions and frameshift mutations or harbor premature stop codons that make them unable to produce functional proteins [41, 42]. Only 10% of human genes have a pseudogene counterpart, and some have just one pseudogene whereas others have multiple pseudogenes [43]. Many pseudogenes have been implicated in tumor biology. Pseudogenes can regulate the expression of their functional counterparts and play a role in tumor development [44–46]. For example, *PTENP1*, a *PTEN* pseudogene, can regulate the level of *PTEN* in cells and exert a growth-suppressive role [46]. The positive correlation that we observed between expression of pseudogene *PA2G4P*4 and that of *PA2G4* suggests that this pseudogene may also regulate the expression of its functional counterpart. Recent reviews describe the role of pseudogenes in normal cellular function and in diseases [42, 47]. A pan-cancer analysis of pseudogene expression in ~2800 patient samples showed that a significant number of pseudogenes are differentially expressed and their expression can classify the major histological subtypes of endometrial cancer [48]. Quantification of pseudogene expression in 13 cancer and normal tissue types found evidence of a wide-spread expression of pseudogenes in cancers and identified cancer/tissue-specific pseudogene expression patterns [49]. Seven (*PA2G4P4*, *ATP5EP2*, *FTH1P3*, *ANXA2P3*, *ANXA2P1*, *HNRNPA1P33*, and *HSP90B3P*) of the 14 pseudogenes that our analysis revealed as important for pan-cancer classification were previously found to be differentially expressed in various cancers.

Lastly, by comparing the top-ranked discriminative genes from “normal” samples to those from tumor samples, we provide evidence that the top-ranked discriminative genes from the tumor samples likely reflect tumor-specific expression differences rather than simply reflecting expression differences attributable to their underlying tissues of origin.

Sexual dimorphism in cancer prevalence and survival between males and females is well-documented but little understood [15, 16]. To see if gene importance in distinguishing the same tumor types differs between males and females, we also carried out pan-cancer classification on 23 TCGA sex non-specific tumor types separately using samples from males and from females. We found that similar prediction accuracies were obtained in 31 pan-cancer and 23 sex non-specific tumor types in both males and females. While most genes had similar ranking for their contribution to tumor type classification in both genders, 21 of the top 100 genes differed in rank by more than 100 between the genders, suggesting that those genes may differ in importance for distinguishing tumor types between males and females. FOXA1 is a known contributor to sexual dimorphism in liver cancer in mice [22]. Our analysis suggested that *FOXA1* expression is more important for distinguishing sex non-specific tumors types in female tumor samples than in male samples. *FOXA1* had significantly higher expression in HNSC, KIRP, and LIHC from females than from males. *FOXA1* is transcriptionally regulated by ESR1 in liver. It is unclear whether *FOXA1* is also regulated by ESR1 in head and neck and kidney; if it were, FOXA1 would likely also have a role in sexual dimorphism in those tumors. Our analysis also suggested that *BNC1* expression is important for distinguishing sex non-specific tumors in males but not in females. *BNC1* is also a putative ESR1 target as ESR1 was bound in the proximal promoter of *BNC1* in T-47D cell line, raising the possibility that BNC1 may also have a role for sexual dimorphism in liver cancer.

## Conclusion

In conclusion, using RNA-seq gene expression alone, we were able to identify many sets of 20 genes that could correctly classify more than 90% of the samples from 31 different tumor types in a validation set. This accuracy is remarkable given the number of the tumor types and the total number of samples involved. This result was largely replicated when we analyzed 23 non-sex-specific tumor types separately for males and females. Genes appearing in the sets of 20 largely overlapped among sets. We regard the frequency with which a gene appeared in those sets as measuring its importance for tumor classification. One third of the 50 most frequently appearing genes were pseudogenes; the degree of enrichment may be indicative of their importance in tumor classification. Lastly, we identified a few genes that might play a role in sexual dimorphism in certain cancers.

## Additional files

Additional file 1: Table S1. “Normal” (normal-adjacent-to-tumor) tissue types and number of TCGA RNA-seq samples used in the analysis. (DOCX 18 kb)

Additional file 2: Table S2. Hyper-parameters used for XGBoost. (DOCX 196 kb)

Additional file 3: Table S3. Schematic of proportion of times samples in the test set were assigned to each of the 31 tumor types and the category of “unclassifiable” across 1000 GA/KNN runs for each of two training/testing partitions (2000 runs total). Only four tumor types (ACC, BLCA, BRCA, and UVM) are shown with sample names denoted generically as S_1_ through Sn, where *n* is the number of samples available for that tumor type. The column containing the proportion correctly classified (π_cc_) is shown in boldface. (DOCX 21 kb)

Additional file 4: Figure S1. Scatterplots of expression levels of *PA2G4* and *PA2G4P4* across all tumor types. (DOCX 383 kb)

Additional file 5: **Methods.** (DOCX 568 kb)

Additional file 6: Figure S2. Heatmap representation of the expression patterns of the top 50 genes across all (a) ACC, (b) BLCA, (c) BRCA, (d) KIRC, (e) KIRP, (f) LGG, and (g) PAAD samples. See Fig. 3 legend for details. The colors of the horizontal bar represent the subgroups identified by *k*-means clustering analysis. (DOCX 60 kb)

Additional file 7: Figure S3. Post-procurement survival probability for patients in the three subtypes of (a) ACC, (b) BLCA, (c) BRCA, (d) KIRC, (e) KIRP, (f) LGG, and (g) PAAD tumors identified by *k*-means analysis based on RNA-seq expression data of the top 50 genes. (DOCX 15 kb)

Additional file 8: Figure S4. Heatmap representation of the expression patterns of the top 50 genes across all 602 “normal” samples taken adjacent to tumors from 17 tumor types. Each row (gene) was centered by the median expression value across all samples. A hierarchical clustering analysis was carried out for both samples and genes using the Euclidean distance as the similarity metric. (DOCX 16 kb)

Additional file 9: Figure S5. Classification accuracies between GA/KNN and XGBoost for 10 testing sets. (DOCX 560 kb)

Additional file 10: Figure S6. Heatmap representation of the expression patterns of the top 50 genes selected by XGBoost across all 602 “normal” samples taken adjacent to tumors from 17 tumor types. Each row (gene) was centered by the median expression value across all samples. A hierarchical clustering analysis was carried out for both samples and genes using the Euclidean distance as the similarity metric. (DOCX 15 kb)

Additional file 11: Figure S7. Proportion of test-set samples predicted to be each of the 23 sex non-specific tumor types in male patients. Y-axis lists the 23 actual tumor types; X-axis lists the 24 possible classification categories (23 tumor types plus “unclassified” [UC]). Each bar represents one of the 24 proportions that samples from the actual tumor type were predicted to be. The 24 plotted proportions represent averages from the corresponding proportions for all samples of the actual tumor type. (DOCX 1745 kb)

Additional file 12: Table S4. Genes ranked among the top 100 from either females and males. (DOCX 389 kb)

Additional file 13: Figure S8. Heatmap representations of the expression patterns of the top genes across all male and female samples. Each row (gene) was centered by the median expression value across all samples. A hierarchical clustering analysis was carried out for both samples and genes using the Euclidean distance as the similarity metric. (DOCX 15 kb)

Additional file 14: Figure S9. Boxplot *BNC1* expression data in the 23 sex non-specific tumors from males (blue) and females (pink). (DOCX 126 kb)

Additional file 15: Table S5. Mean and median of for π_cc_ values for each tumor type from full female dataset, full male dataset, and the corresponding mean (sd) from the eight “matched” male datasets. (DOCX 283 kb)

## Abbreviations

GA/KNN: Genetic algorithm/*k*-nearest neighbors
TCGA: The Cancer Genome Atlas
