# Supplementary material for: A comprehensive genomic pan-cancer classification using The Cancer Genome Atlas gene expression data
Source: BMC Genomics. 2017 Jul 3;18:508. doi: 10.1186/s12864-017-3906-0 (PMC5496318; doi:10.1186/s12864-017-3906-0)
Supplement: Supplementary file 1 — “Normal” (normal-adjacent-to-tumor) tissue types and number of TCGA RNA-seq samples used in the analysis. (DOCX 18 kb) [file 12864_2017_3906_MOESM3_ESM.docx]

**Additional file 3: Table S3 for**

**A comprehensive genomic pan-cancer classification using The Cancer Genome Atlas gene expression data**

**Table S3**. Schematic of proportion of times samples in the test set were assigned to each of the 31 tumor types and the category of “unclassifiable” across 1,000 GA/KNN runs for each of two training/testing partitions (2,000 runs total). Only four tumor types (ACC, BLCA, BRCA, and UVM) are shown with sample names denoted generically as S1 through S*n*, where *n* is the number of samples available for that tumor type. The column containing the proportion correctly classified (π_cc_) is shown in boldface.

| Type | Sample | ACC | BLCA | BRCA | CESC | ∙∙∙ | THYM | UCS | UVM | Unclassifiable |
| --- | --- | --- | --- | --- | --- | --- | --- | --- | --- | --- |
| ACC | S1 | **0.911** | 0.000 | 0.002 | 0.000 | ∙∙∙ | 0.000 | 0.000 | 0.000 | 0.041 |
|  | S2 | **0.938** | 0.000 | 0.001 | 0.000 | ∙∙∙ | 0.000 | 0.000 | 0.000 | 0.028 |
|  | S3 | **0.885** | 0.000 | 0.000 | 0.000 | ∙∙∙ | 0.000 | 0.000 | 0.001 | 0.038 |
|  | S4 | **0.946** | 0.000 | 0.000 | 0.000 | ∙∙∙ | 0.000 | 0.000 | 0.000 | 0.019 |
|  | ⁞ | ⁞ | ⁞ | ⁞ | ⁞ | ⁞ | ⁞ | ⁞ | ⁞ | ⁞ |
|  | S79 | **0.232** | 0.001 | 0.006 | 0.000 | ∙∙∙ | 0.000 | 0.000 | 0.004 | 0.304 |
| BLCA | S1 | 0.000 | **0.823** | 0.001 | 0.108 | ∙∙∙ | 0.000 | 0.000 | 0.000 | 0.048 |
|  | S2 | 0.000 | **0.138** | 0.196 | 0.014 | ∙∙∙ | 0.007 | 0.000 | 0.000 | 0.376 |
|  | S3 | 0.000 | **0.986** | 0.000 | 0.000 | ∙∙∙ | 0.001 | 0.000 | 0.000 | 0.011 |
|  | S4 | 0.009 | **0.041** | 0.006 | 0.005 | ∙∙∙ | 0.001 | 0.000 | 0.002 | 0.531 |
|  | ⁞ | ⁞ | ⁞ | ⁞ | ⁞ | ⁞ | ⁞ | ⁞ | ⁞ | ⁞ |
|  | S408 | 0.000 | 0.983 | 0.000 | 0.002 | ∙∙∙ | 0.000 | 0.000 | 0.000 | 0.008 |
| BRCA | S1 | 0.000 | 0.000 | **1.000** | 0.000 | ∙∙∙ | 0.000 | 0.000 | 0.000 | 0.000 |
|  | S2 | 0.000 | 0.000 | **1.000** | 0.000 | ∙∙∙ | 0.000 | 0.000 | 0.000 | 0.000 |
|  | S3 | 0.000 | 0.004 | **0.978** | 0.000 | ∙∙∙ | 0.001 | 0.000 | 0.000 | 0.007 |
|  | S4 | 0.000 | 0.001 | **0.986** | 0.000 | ∙∙∙ | 0.001 | 0.000 | 0.000 | 0.006 |
|  | ⁞ | ⁞ | ⁞ | ⁞ | ⁞ | ⁞ | ⁞ | ⁞ | ⁞ | ⁞ |
|  | S1102 | 0.000 | 0.004 | **0.979** | 0.000 | ∙∙∙ | 0.001 | 0.000 | 0.000 | 0.011 |
|  | ⁞ | ⁞ | ⁞ | ⁞ | ⁞ | ⁞ | ⁞ | ⁞ | ⁞ | ⁞ |
| UVM | S1 | 0.000 | 0.000 | 0.000 | 0.000 | ∙∙∙ | 0.000 | 0.000 | **0.982** | 0.000 |
|  | S2 | 0.000 | 0.000 | 0.000 | 0.000 | ∙∙∙ | 0.000 | 0.000 | **1.000** | 0.000 |
|  | S3 | 0.000 | 0.000 | 0.000 | 0.000 | ∙∙∙ | 0.000 | 0.000 | **0.999** | 0.000 |
|  | S4 | 0.000 | 0.000 | 0.000 | 0.000 | ∙∙∙ | 0.000 | 0.000 | **0.970** | 0.001 |
|  | ⁞ | ⁞ | ⁞ | ⁞ | ⁞ | ⁞ | ⁞ | ⁞ | ⁞ | ⁞ |
|  | S80 | 0.000 | 0.000 | 0.000 | 0.000 | ∙∙∙ | 0.000 | 0.000 | **0.993** | 0.000 |
