# Supplementary material for: A comprehensive genomic pan-cancer classification using The Cancer Genome Atlas gene expression data
Source: BMC Genomics. 2017 Jul 3;18:508. doi: 10.1186/s12864-017-3906-0 (PMC5496318; doi:10.1186/s12864-017-3906-0)
Supplement: Supplementary file 2 — Hyper-parameters used for XGBoost. (DOCX 196 kb) [file 12864_2017_3906_MOESM11_ESM.docx]

**Additional file 11: Figure S7 for**

**A comprehensive genomic pan-cancer classification using The Cancer Genome Atlas gene expression data**


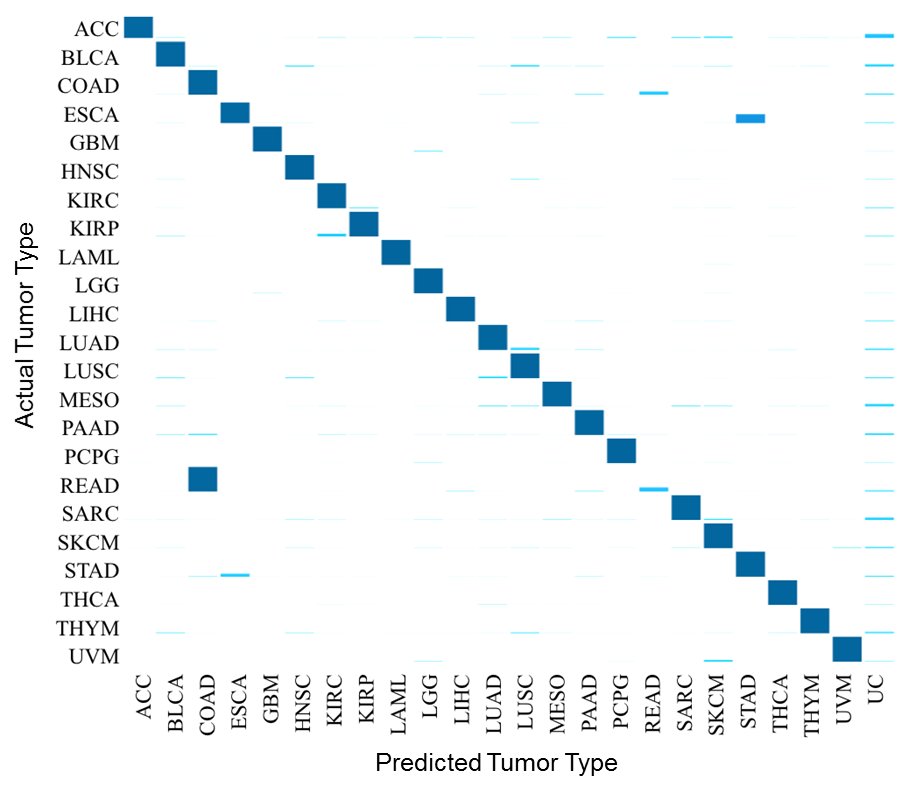


1. **Male sex non-specific tumor types**


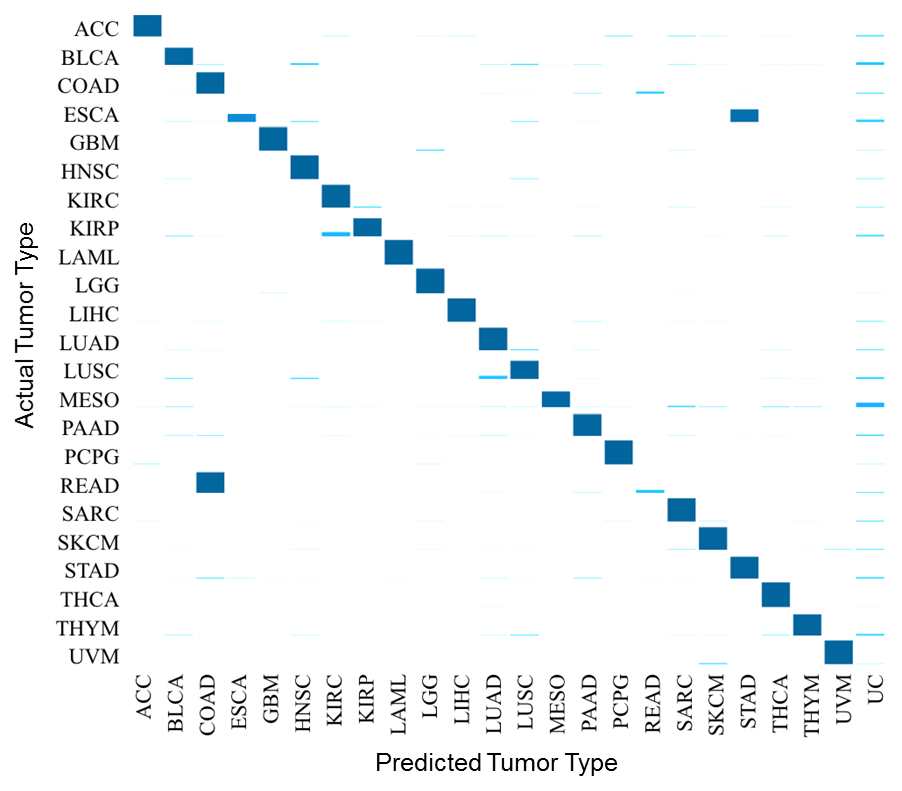


1. **Female sex non-specific tumor types**

**Figure S7** Proportion of test-set samples predicted to be each of the 23 sex non-specific tumor types in male patients. Y-axis lists the 23 actual tumor types; x-axis lists the 24 possible classification categories (23 tumor types plus “unclassified” [UC]). Each bar represents one of the 24 proportions that samples from the actual tumor type were predicted to be. The 24 plotted proportions represent averages from the corresponding proportions for all samples of the actual tumor type.
