# Supplementary material for: A comprehensive genomic pan-cancer classification using The Cancer Genome Atlas gene expression data
Source: BMC Genomics. 2017 Jul 3;18:508. doi: 10.1186/s12864-017-3906-0 (PMC5496318; doi:10.1186/s12864-017-3906-0)
Supplement: Supplementary file 3 — Schematic of proportion of times samples in the test set were assigned to each of the 31 tumor types and the category of “unclassifiable” across 1000 GA/KNN runs for each of two training/testing partitions (2000 runs total). Only four tumor types (ACC, BLCA, BRCA, and UVM) are shown with sample names denoted generically as S1 through Sn, where n is the number of samples available for that tumor type. The column containing the proportion correctly classified (πcc) is shown in boldface. (DOCX 21 kb) [file 12864_2017_3906_MOESM8_ESM.docx]

**Additional file 8: Table S4 for**

**A comprehensive genomic pan-cancer classification using The Cancer Genome Atlas gene expression data**

**Table S4.** Genes ranked among the top 100 from either females and males.

| **Gene** | **ID** | **Rank from Females** | **Rank from Males** | **Difference (F-M)** |
| --- | --- | --- | --- | --- |
| FTH1P3 | 2498 | 1 | 1 | 0 |
| HSPB1P1 | 653553 | 2 | 3 | -1 |
| PA2G4P4 | 647033 | 3 | 2 | 1 |
| GJB1 | 2705 | 4 | 28 | -24 |
| LOC644936 | 644936 | 5 | 7 | -2 |
| NAPSA | 9476 | 6 | 17 | -11 |
| SFTA3 | 253970 | 7 | 20 | -13 |
| LOC643387 | 643387 | 8 | 14 | -6 |
| NACAP1 | 83955 | 9 | 10 | -1 |
| EEF1A1P9 | 441032 | 10 | 8 | 2 |
| ANXA2P3 | 305 | 11 | 4 | 7 |
| HNF1B | 6928 | 12 | 19 | -7 |
| UQCRBP1 | 442454 | 13 | 16 | -3 |
| SLC34A2 | 10568 | 14 | 71 | -57 |
| EPCAM | 4072 | 15 | 43 | -28 |
| SFTPB | 6439 | 16 | 13 | 3 |
| MFSD2B | 388931 | 17 | 15 | 2 |
| HAND2 | 9464 | 18 | 32 | -14 |
| IGBP1P1 | 280655 | 19 | 18 | 1 |
| PPIAL4C | 653598 | 20 | 26 | -6 |
| PTTG3P | 26255 | 21 | 6 | 15 |
| MAB21L2 | 10586 | 22 | 46 | -24 |
| ATP5EP2 | 432369 | 23 | 9 | 14 |
| NKX2-1 | 7080 | 24 | 37 | -13 |
| YBX3P1 | 440359 | 25 | 24 | 1 |
| SFTPD | 6441 | 26 | 30 | -4 |
| PABPC3 | 5042 | 27 | 33 | -6 |
| SFTPA1 | 653509 | 28 | 12 | 16 |
| CELSR2 | 1952 | 29 | 84 | -55 |
| RPL19P12 | 100129424 | 30 | 21 | 9 |
| NME2P1 | 283458 | 31 | 36 | -5 |
| SFTPC | 6440 | 32 | 35 | -3 |
| HNRNPA3P1 | 10151 | 33 | 40 | -7 |
| NACA2 | 342538 | 34 | 11 | 23 |
| HNF4A | 3172 | 35 | 60 | -25 |
| HOXA9 | 3205 | 36 | 56 | -20 |
| ERBB3 | 2065 | 37 | 79 | -42 |
| H3F3C | 440093 | 38 | 23 | 15 |
| RPL23P8 | 222901 | 39 | 22 | 17 |
| TBX5 | 6910 | 40 | 5 | 35 |
| SCGB3A2 | 117156 | 41 | 68 | -27 |
| SOX10 | 6663 | 42 | 118 | -76 |
| ANXA2P1 | 303 | 43 | 29 | 14 |
| HSP90B3P | 343477 | 44 | 34 | 10 |
| LAD1 | 3898 | 45 | 269 | -224 |
| HNF1A-AS1 | 283460 | 46 | 62 | -16 |
| HNRNPA1P33 | 728643 | 47 | 66 | -19 |
| RPL17 | 6139 | 48 | 27 | 21 |
| HNRNPCL3 | 649330 | 49 | 38 | 11 |
| TP63 | 8626 | 50 | 25 | 25 |
| FXYD3 | 5349 | 51 | 147 | -96 |
| TCF21 | 6943 | 52 | 41 | 11 |
| ELF3 | 1999 | 53 | 127 | -74 |
| HNF1A | 6927 | 54 | 80 | -26 |
| TMC5 | 79838 | 55 | 241 | -186 |
| HAND2-AS1 | 79804 | 56 | 72 | -16 |
| ZBTB20 | 26137 | 57 | 131 | -74 |
| CSNK1A1L | 122011 | 58 | 42 | 16 |
| CTAGE1 | 64693 | 59 | 49 | 10 |
| KIF4B | 285643 | 60 | 55 | 5 |
| MALAT1 | 378938 | 61 | 88 | -27 |
| GAL3ST1 | 9514 | 62 | 76 | -14 |
| ZNF750 | 79755 | 63 | 87 | -24 |
| GGTLC1 | 92086 | 64 | 99 | -35 |
| FUNDC2P2 | 388965 | 65 | 61 | 4 |
| SLC3A1 | 6519 | 66 | 154 | -88 |
| AQP4 | 361 | 67 | 128 | -61 |
| SFTPA2 | 729238 | 68 | 31 | 37 |
| TMEM125 | 128218 | 69 | 85 | -16 |
| PRR15L | 79170 | 70 | 155 | -85 |
| TBX3 | 6926 | 71 | 73 | -2 |
| POTEM | 641455 | 72 | 89 | -17 |
| ABCC6 | 368 | 73 | 52 | 21 |
| HPN | 3249 | 74 | 336 | -262 |
| ITGB6 | 3694 | 75 | 148 | -73 |
| SH3TC2 | 79628 | 76 | 119 | -43 |
| SND1-IT1 | 27099 | 77 | 59 | 18 |
| CLK2P1 | 1197 | 78 | 77 | 1 |
| STK32A | 202374 | 79 | 226 | -147 |
| GATA6 | 2627 | 80 | 75 | 5 |
| CFAP221 | 200373 | 81 | 187 | -106 |
| FOXA1 | 3169 | 82 | 417 | -335 |
| PDZK1 | 5174 | 83 | 293 | -210 |
| HOXA11 | 3207 | 84 | 184 | -100 |
| NTF4 | 4909 | 85 | 69 | 16 |
| TRIM29 | 23650 | 86 | 188 | -102 |
| HOXA11-AS | 221883 | 87 | 139 | -52 |
| CLDN4 | 1364 | 88 | 83 | 5 |
| KIF12 | 113220 | 89 | 249 | -160 |
| MIR205HG | 642587 | 90 | 39 | 51 |
| ANP32AP1 | 723972 | 91 | 44 | 47 |
| KRT7 | 3855 | 92 | 103 | -11 |
| LOC341056 | 341056 | 93 | 64 | 29 |
| SMIM6 | 100130933 | 94 | 78 | 16 |
| FERMT1 | 55612 | 95 | 177 | -82 |
| PTPRZ1 | 5803 | 96 | 109 | -13 |
| WBP11P1 | 441818 | 97 | 57 | 40 |
| LOC407835 | 407835 | 98 | 58 | 40 |
| SLC44A4 | 80736 | 99 | 140 | -41 |
| AMY1A | 276 | 100 | 370 | -270 |
| SFN | 2810 | 102 | 91 | 11 |
| HGD | 3081 | 106 | 81 | 25 |
| MYRF | 745 | 107 | 50 | 57 |
| GCNT2 | 2651 | 117 | 82 | 35 |
| LOC100130331 | 100130331 | 120 | 54 | 66 |
| ANXA8L2 | 244 | 122 | 63 | 59 |
| GCSH | 2653 | 124 | 95 | 29 |
| GATA3-AS1 | 399717 | 126 | 51 | 75 |
| PVRL1 | 5818 | 128 | 96 | 32 |
| PLS1 | 5357 | 134 | 70 | 64 |
| HOXD8 | 3234 | 138 | 86 | 52 |
| KRT18 | 3875 | 139 | 67 | 72 |
| GATA3 | 2625 | 141 | 53 | 88 |
| FAM83B | 222584 | 149 | 92 | 57 |
| IRF6 | 3664 | 150 | 74 | 76 |
| ANXA8 | 653145 | 161 | 48 | 113 |
| KRT8 | 3856 | 175 | 65 | 110 |
| CLRN3 | 119467 | 204 | 98 | 106 |
| CSTA | 1475 | 218 | 93 | 125 |
| ANKS4B | 257629 | 245 | 97 | 148 |
| S1PR5 | 53637 | 281 | 100 | 181 |
| RNF43 | 54894 | 299 | 94 | 205 |
| KRT5 | 3852 | 328 | 47 | 281 |
| FAT2 | 2196 | 392 | 90 | 302 |
| BNC1 | 646 | 932 | 45 | 887 |
