# Supplementary material for: A comprehensive genomic pan-cancer classification using The Cancer Genome Atlas gene expression data
Source: BMC Genomics. 2017 Jul 3;18:508. doi: 10.1186/s12864-017-3906-0 (PMC5496318; doi:10.1186/s12864-017-3906-0)
Supplement: Supplementary file 4 — Scatterplots of expression levels of PA2G4 and PA2G4P4 across all tumor types. (DOCX 383 kb) [file 12864_2017_3906_MOESM10_ESM.docx]

**Additional file 10: Figure S6 for**

**A comprehensive genomic pan-cancer classification using The Cancer Genome Atlas gene expression data**


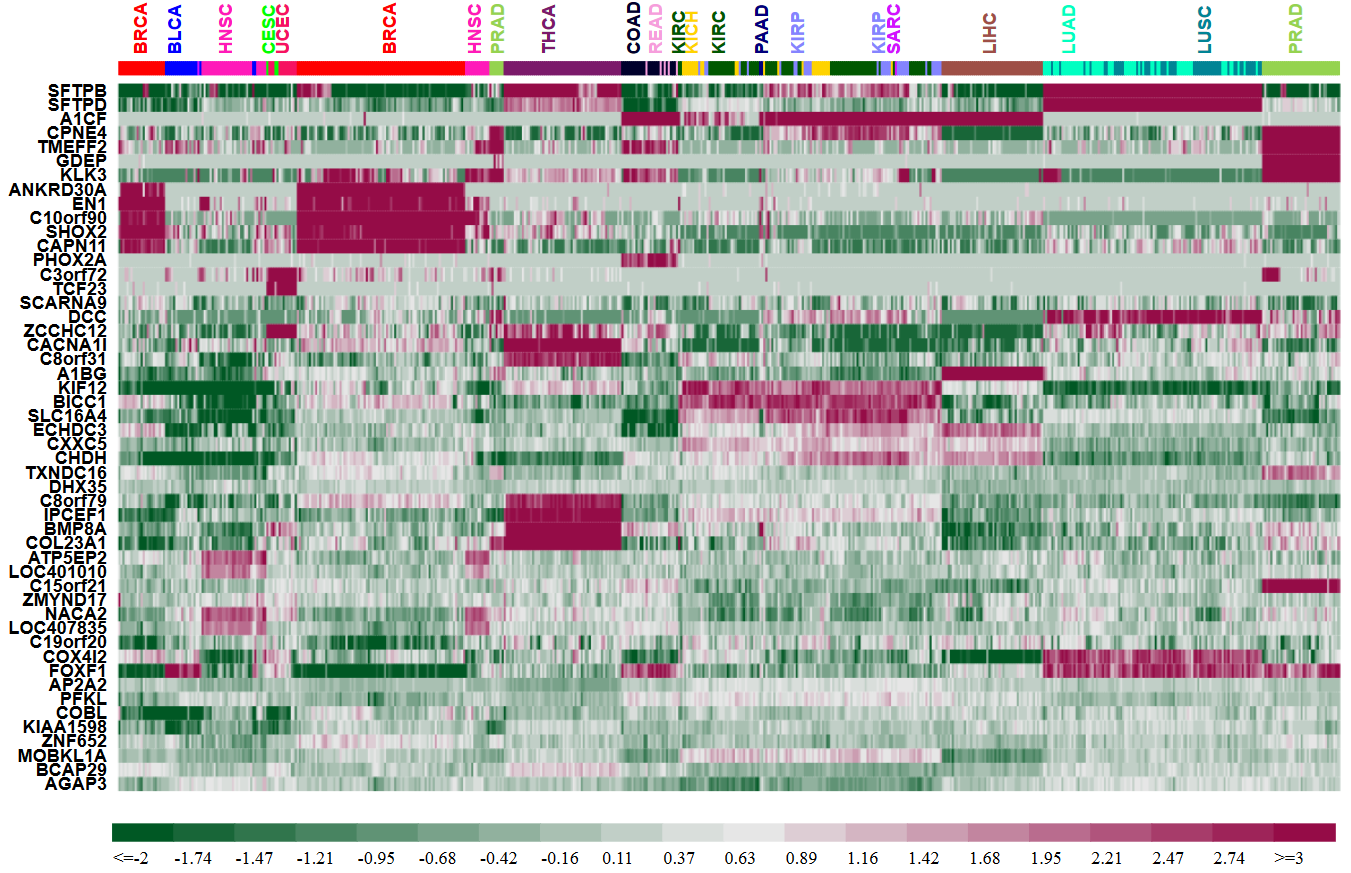


**Figure S6** Heatmap representation of the expression patterns of the top 50 genes selected by XGBoost across all 602 “normal” samples taken adjacent to tumors from 17 tumor types. Each row (gene) was centered by the median expression value across all samples. A hierarchical clustering analysis was carried out for both samples and genes using the Euclidean distance as the similarity metric.
