# Supplementary material for: A comprehensive genomic pan-cancer classification using The Cancer Genome Atlas gene expression data
Source: BMC Genomics. 2017 Jul 3;18:508. doi: 10.1186/s12864-017-3906-0 (PMC5496318; doi:10.1186/s12864-017-3906-0)
Supplement: Supplementary file 5 — Methods. (DOCX 568 kb) [file 12864_2017_3906_MOESM13_ESM.docx]

**Additional file 13: Figure S8 for**

**A comprehensive genomic pan-cancer classification using The Cancer Genome Atlas gene expression data**

| 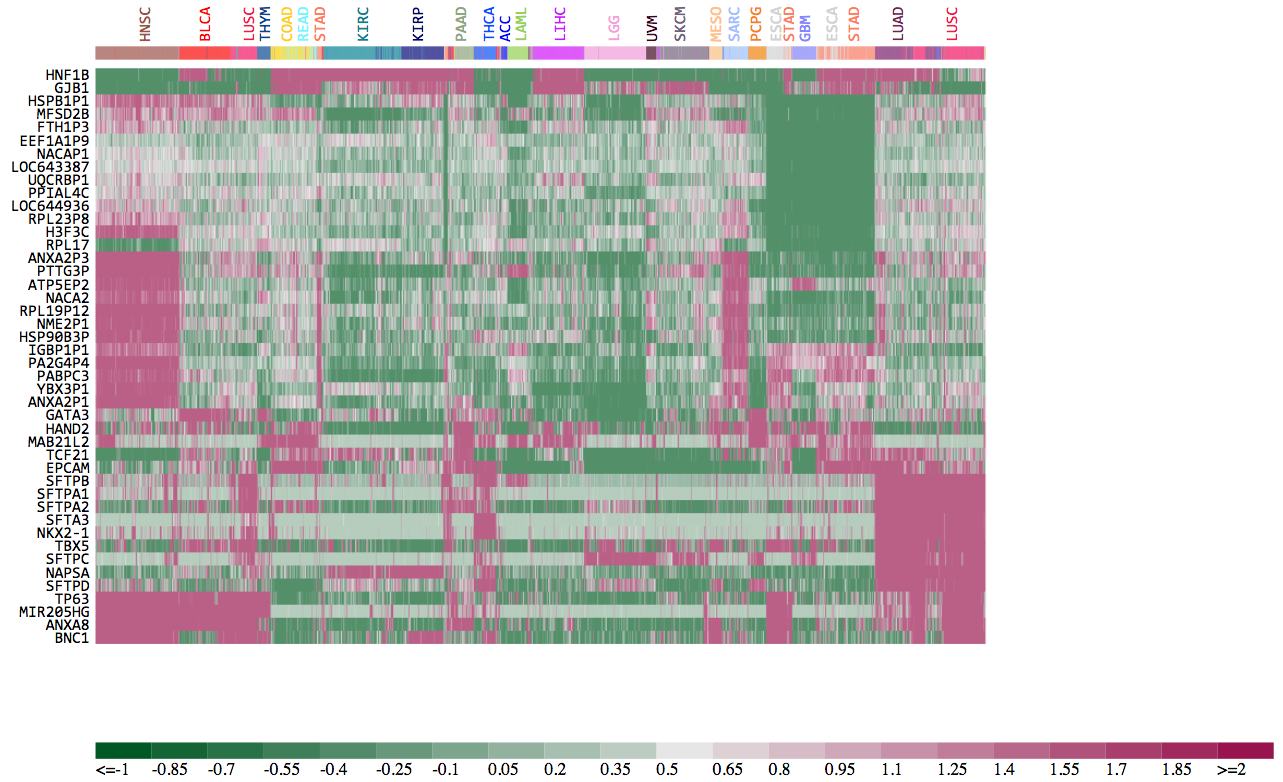 | 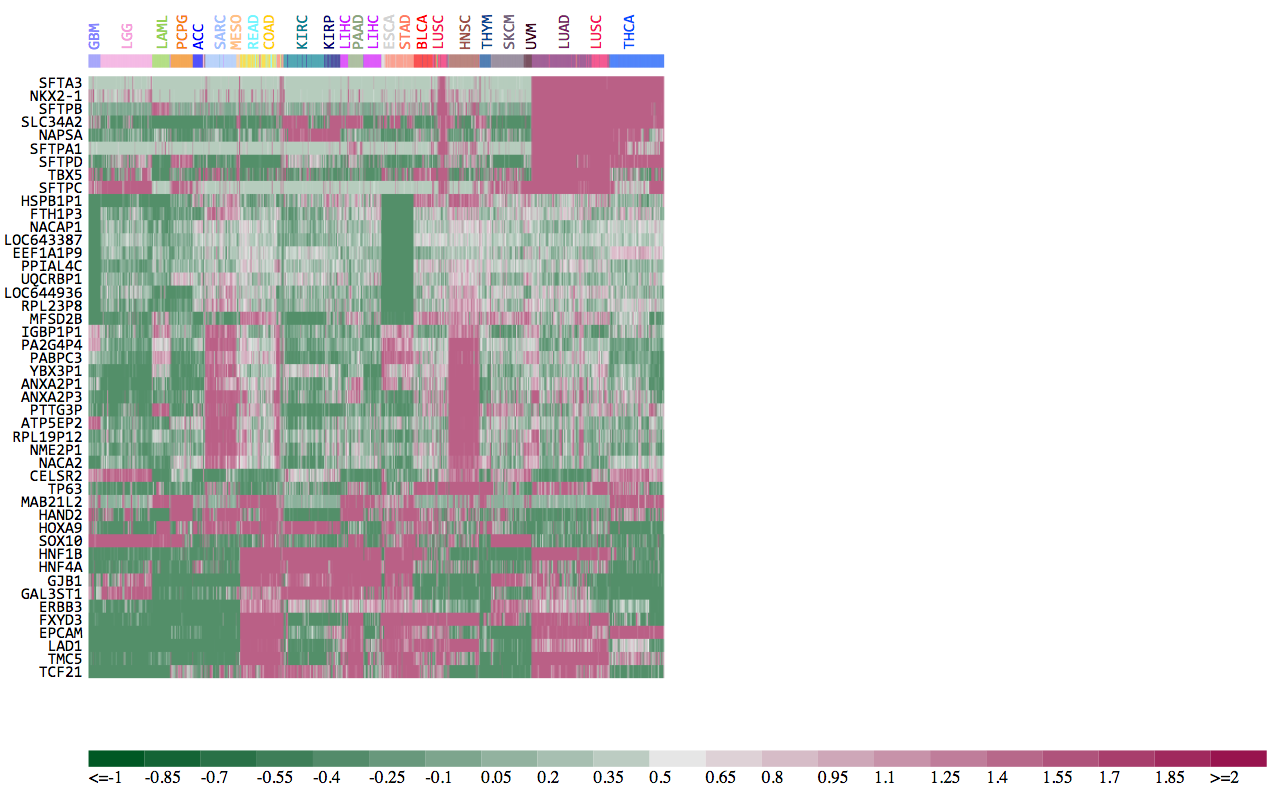 |
| --- | --- |
| 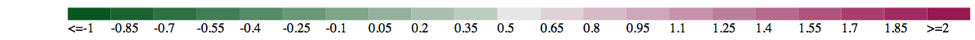 | |
| **(a)** Top 44 genes across all 4,081 male samples | 1. Top 46 genes across all 2,638 female samples |

**Figure S8** Heatmap representations of the expression patterns of the top genes across all male and female samples. Each row (gene) was centered by the median expression value across all samples. A hierarchical clustering analysis was carried out for both samples and genes using the Euclidean distance as the similarity metric.
