# Supplementary material for: A comprehensive genomic pan-cancer classification using The Cancer Genome Atlas gene expression data
Source: BMC Genomics. 2017 Jul 3;18:508. doi: 10.1186/s12864-017-3906-0 (PMC5496318; doi:10.1186/s12864-017-3906-0)
Supplement: Supplementary file 6 — Heatmap representation of the expression patterns of the top 50 genes across all (a) ACC, (b) BLCA, (c) BRCA, (d) KIRC, (e) KIRP, (f) LGG, and (g) PAAD samples. See Fig. 3 legend for details. The colors of the horizontal bar represent the subgroups identified by k-means clustering analysis. (DOCX 60 kb) [file 12864_2017_3906_MOESM14_ESM.docx]

**Additional file 14: Figure S9 for**

**A comprehensive genomic pan-cancer classification using The Cancer Genome Atlas gene expression data**


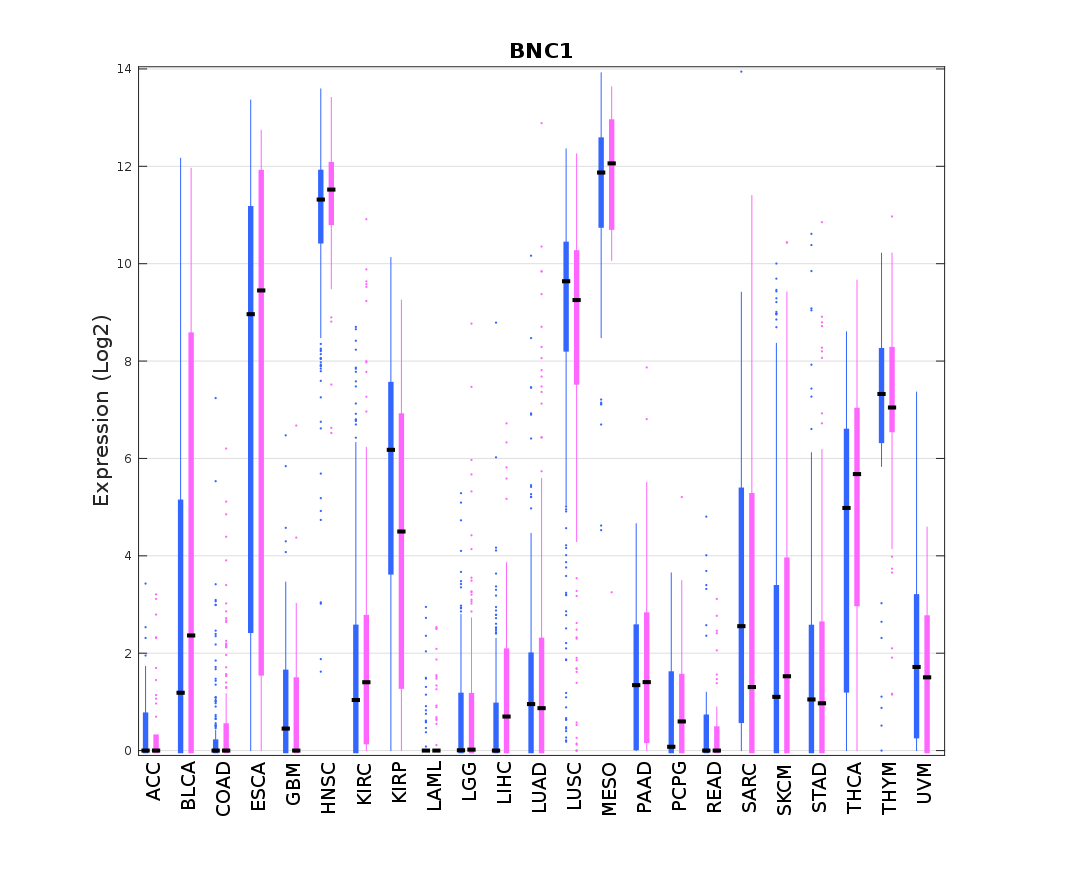


**Figure S9** Boxplot *BNC1* expression data in the 23 sex non-specific tumors from males (blue) and females (pink).
