# Supplementary material for: A comprehensive genomic pan-cancer classification using The Cancer Genome Atlas gene expression data
Source: BMC Genomics. 2017 Jul 3;18:508. doi: 10.1186/s12864-017-3906-0 (PMC5496318; doi:10.1186/s12864-017-3906-0)
Supplement: Supplementary file 7 — Post-procurement survival probability for patients in the three subtypes of (a) ACC, (b) BLCA, (c) BRCA, (d) KIRC, (e) KIRP, (f) LGG, and (g) PAAD tumors identified by k-means analysis based on RNA-seq expression data of the top 50 genes. (DOCX 15 kb) [file 12864_2017_3906_MOESM9_ESM.docx]

**Additional file 9: Table S5 for**

**A comprehensive genomic pan-cancer classification using The Cancer Genome Atlas gene expression data**

**Table S5**. Mean and median of for π_cc_ values for each tumor type from full female dataset, full male dataset, and the corresponding mean (sd) from the eight “matched” male datasets.

| Type | Mean | | | Median | | |
| --- | --- | --- | --- | --- | --- | --- |
|  | Female full dataset | Male full dataset | Mean(sd) from 8 matched male datasets | Female full dataset | Male full dataset | Median(sd) from 8 matched male datasets |
| ACC | 0.83 | 0.68 | 0.75 (0.02) | 0.89 | 0.71 | 0.79 (0.02) |
| BLCA | 0.67 | 0.77 | 0.66 (0.02) | 0.77 | 0.86 | 0.73 (0.03) |
| COAD | 0.86 | 0.84 | 0.84 (0.01) | 0.89 | 0.87 | 0.87 (0.01) |
| GBM | 0.92 | 0.96 | 0.94 (0.01) | 0.97 | 0.99 | 0.97 (0.01) |
| HNSC | 0.94 | 0.95 | 0.92 (0.00) | 0.99 | 0.99 | 0.96 (0.01) |
| KIRC | 0.92 | 0.94 | 0.93 (0.01) | 1.00 | 1.00 | 1.00 (0.00) |
| KIRP | 0.69 | 0.87 | 0.82 (0.02) | 0.74 | 0.99 | 0.96 (0.01) |
| LAML | 1.00 | 0.99 | 0.99 (0.00) | 1.00 | 1.00 | 1.00 (0.00) |
| LGG | 0.99 | 0.98 | 0.99 (0.01) | 1.00 | 1.00 | 1.00 (0.00) |
| LIHC | 0.93 | 0.95 | 0.94 (0.01) | 0.98 | 1.00 | 0.99 (0.00) |
| LUAD | 0.90 | 0.84 | 0.88 (0.01) | 0.98 | 0.92 | 0.95 (0.00) |
| LUSC | 0.71 | 0.86 | 0.78 (0.02) | 0.87 | 0.95 | 0.87 (0.02) |
| MESO | 0.63 | 0.81 | 0.48 (0.08) | 0.70 | 0.89 | 0.49 (0.10) |
| PAAD | 0.86 | 0.86 | 0.87 (0.02) | 0.96 | 0.94 | 0.96 (0.01) |
| PCPG | 0.95 | 0.97 | 0.98 (0.00) | 0.99 | 0.99 | 0.99 (0.00) |
| READ | 0.12 | 0.13 | 0.14 (0.01) | 0.11 | 0.13 | 0.14 (0.01) |
| SARC | 0.91 | 0.84 | 0.85 (0.01) | 0.95 | 0.90 | 0.93 (0.00) |
| SKCM | 0.90 | 0.90 | 0.89 (0.01) | 0.96 | 0.97 | 0.95 (0.01) |
| THCA | 0.99 | 0.97 | 0.98 (0.00) | 1.00 | 0.99 | 1.00 (0.01) |
| THYM | 0.83 | 0.87 | 0.89 (0.01) | 0.96 | 0.98 | 0.98 (0.01) |
| UVM | 0.97 | 0.92 | 0.93 (0.01) | 0.99 | 0.97 | 0.97 (0.01) |
| ESCA | 0.30 | 0.65 | 0.41 (0.13) | 0.17 | 0.74 | 0.34 (0.32) |
| STAD | 0.86 | 0.85 | 0.89 (0.01) | 0.95 | 0.90 | 0.97 (0.01) |
