# Supplementary material for: A comprehensive genomic pan-cancer classification using The Cancer Genome Atlas gene expression data
Source: BMC Genomics. 2017 Jul 3;18:508. doi: 10.1186/s12864-017-3906-0 (PMC5496318; doi:10.1186/s12864-017-3906-0)
Supplement: Supplementary file 8 — Heatmap representation of the expression patterns of the top 50 genes across all 602 “normal” samples taken adjacent to tumors from 17 tumor types. Each row (gene) was centered by the median expression value across all samples. A hierarchical clustering analysis was carried out for both samples and genes using the Euclidean distance as the similarity metric. (DOCX 16 kb) [file 12864_2017_3906_MOESM1_ESM.docx]

**Additional file 1: Table S1 for**

**A comprehensive genomic pan-cancer classification using The Cancer Genome Atlas gene expression data**

**Table S1.** “Normal” (normal-adjacent-to-tumor) tissue types and number of TCGA RNA-seq samples used in the analysis

| Tumor name | TCGA code | Number of samples |
| --- | --- | --- |
| Bladder urothelial carcinoma | BLCA | 19 |
| Breast invasive carcinoma | BRCA | 106 |
| Cervical squamous cell carcinoma and endocervical adenocarcinoma | CESC | 3 |
| Colon adenocarcinoma | COAD | 23 |
| Head and Neck squamous cell carcinoma | HNSC | 42 |
| Kidney chromophobe | KICH | 25 |
| Kidney renal clear cell carcinoma | KIRC | 72 |
| Kidney renal papillary cell carcinoma | KIRP | 30 |
| Liver hepatocellular carcinoma | LIHC | 50 |
| Lung adenocarcinoma | LUAD | 58 |
| Lung squamous cell carcinoma | LUSC | 50 |
| Pancreatic agenocarcinoma | PAAD | 2 |
| Prostate adenocarcinoma | PRAD | 45 |
| Rectum adenocarcinoma | READ | 5 |
| Sarcoma | SARC | 2 |
| Thyroid carcinoma | THCA | 58 |
| Uterine corpus endometrial carcinoma | UCEC | 12 |
