# Supplementary material for: A comprehensive genomic pan-cancer classification using The Cancer Genome Atlas gene expression data
Source: BMC Genomics. 2017 Jul 3;18:508. doi: 10.1186/s12864-017-3906-0 (PMC5496318; doi:10.1186/s12864-017-3906-0)
Supplement: Supplementary file 9 — Classification accuracies between GA/KNN and XGBoost for 10 testing sets. (DOCX 560 kb) [file 12864_2017_3906_MOESM4_ESM.docx]

**Additional file 4: Figure S1 for**

**A comprehensive genomic pan-cancer classification using The Cancer Genome Atlas gene expression data**

**
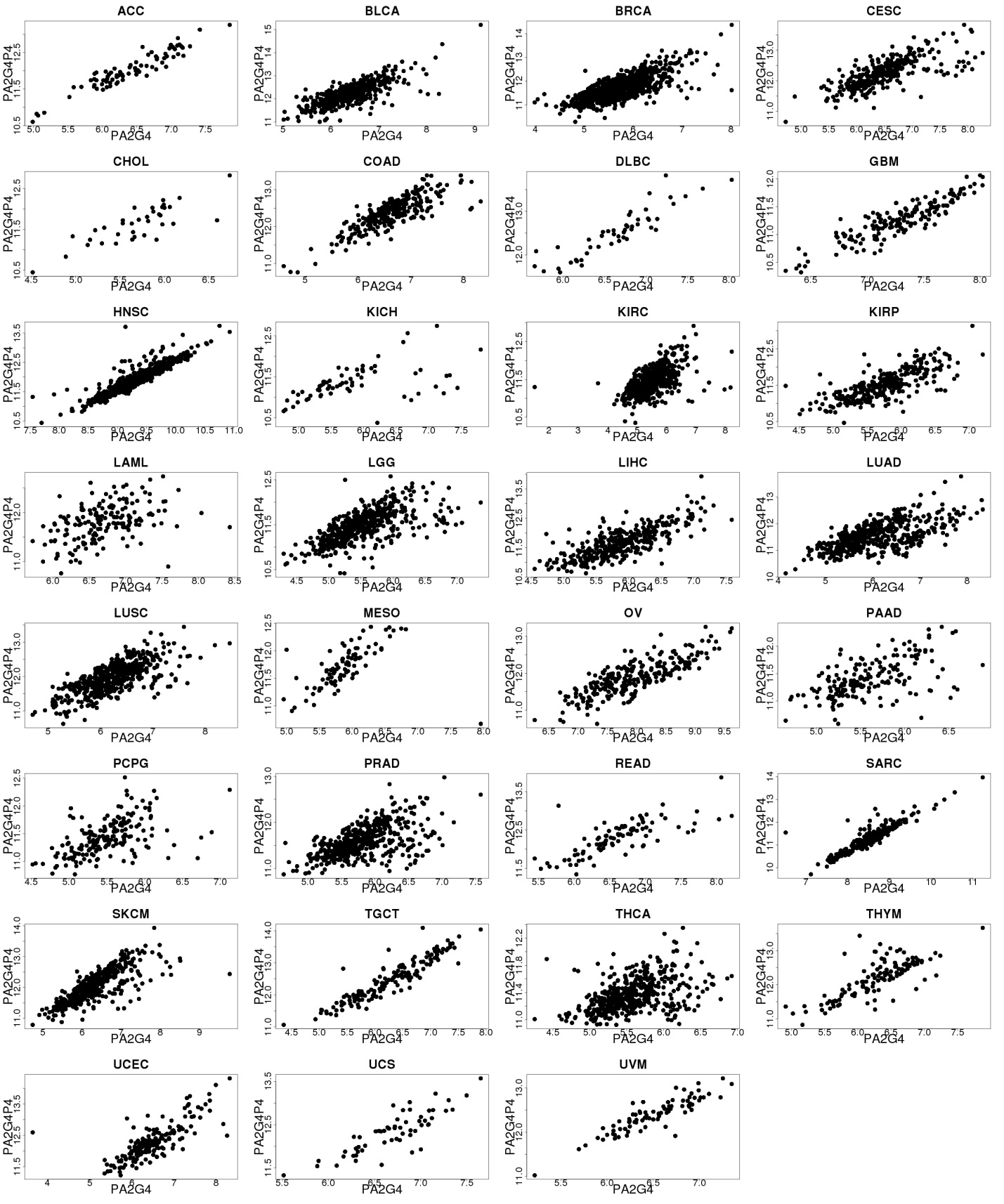
**

**Figure S1** Scatterplots of expression levels of *PA2G4* and *PA2G4P4* across all tumor types.
