# Supplementary material for: A comprehensive genomic pan-cancer classification using The Cancer Genome Atlas gene expression data
Source: BMC Genomics. 2017 Jul 3;18:508. doi: 10.1186/s12864-017-3906-0 (PMC5496318; doi:10.1186/s12864-017-3906-0)
Supplement: Supplementary file 10 — Heatmap representation of the expression patterns of the top 50 genes selected by XGBoost across all 602 “normal” samples taken adjacent to tumors from 17 tumor types. Each row (gene) was centered by the median expression value across all samples. A hierarchical clustering analysis was carried out for both samples and genes using the Euclidean distance as the similarity metric. (DOCX 15 kb) [file 12864_2017_3906_MOESM2_ESM.docx]

**Additional file 2: Table S2 for**

**A comprehensive genomic pan-cancer classification using The Cancer Genome Atlas gene expression data**

**Table S2**. Hyper-parameters used for XGBoost.

| **Hyper-parameter** | **Optimization range** | **Optimal value** |
| --- | --- | --- |
| Learning rate | [0.04 – 0.1] | 0.1 |
| Maximum tree depth | [4, 6, 8, 10] | 4 |
| The minimum child weight | [1 – 10] | 1 |
| Subsample rate (row sampling) | [0.5 – 1] | 0.75 |
| % feature used in each boost (column sampling) | [0.4 – 1] | 0.75 |
