# Supplementary material for: A comprehensive genomic pan-cancer classification using The Cancer Genome Atlas gene expression data
Source: BMC Genomics. 2017 Jul 3;18:508. doi: 10.1186/s12864-017-3906-0 (PMC5496318; doi:10.1186/s12864-017-3906-0)
Supplement: Supplementary file 11 — Proportion of test-set samples predicted to be each of the 23 sex non-specific tumor types in male patients. Y-axis lists the 23 actual tumor types; X-axis lists the 24 possible classification categories (23 tumor types plus “unclassified” [UC]). Each bar represents one of the 24 proportions that samples from the actual tumor type were predicted to be. The 24 plotted proportions represent averages from the corresponding proportions for all samples of the actual tumor type. (DOCX 1745 kb) [file 12864_2017_3906_MOESM6_ESM.docx]

**Additional file 6: Figure S2 for**

**A comprehensive genomic pan-cancer classification using The Cancer Genome Atlas gene expression data**


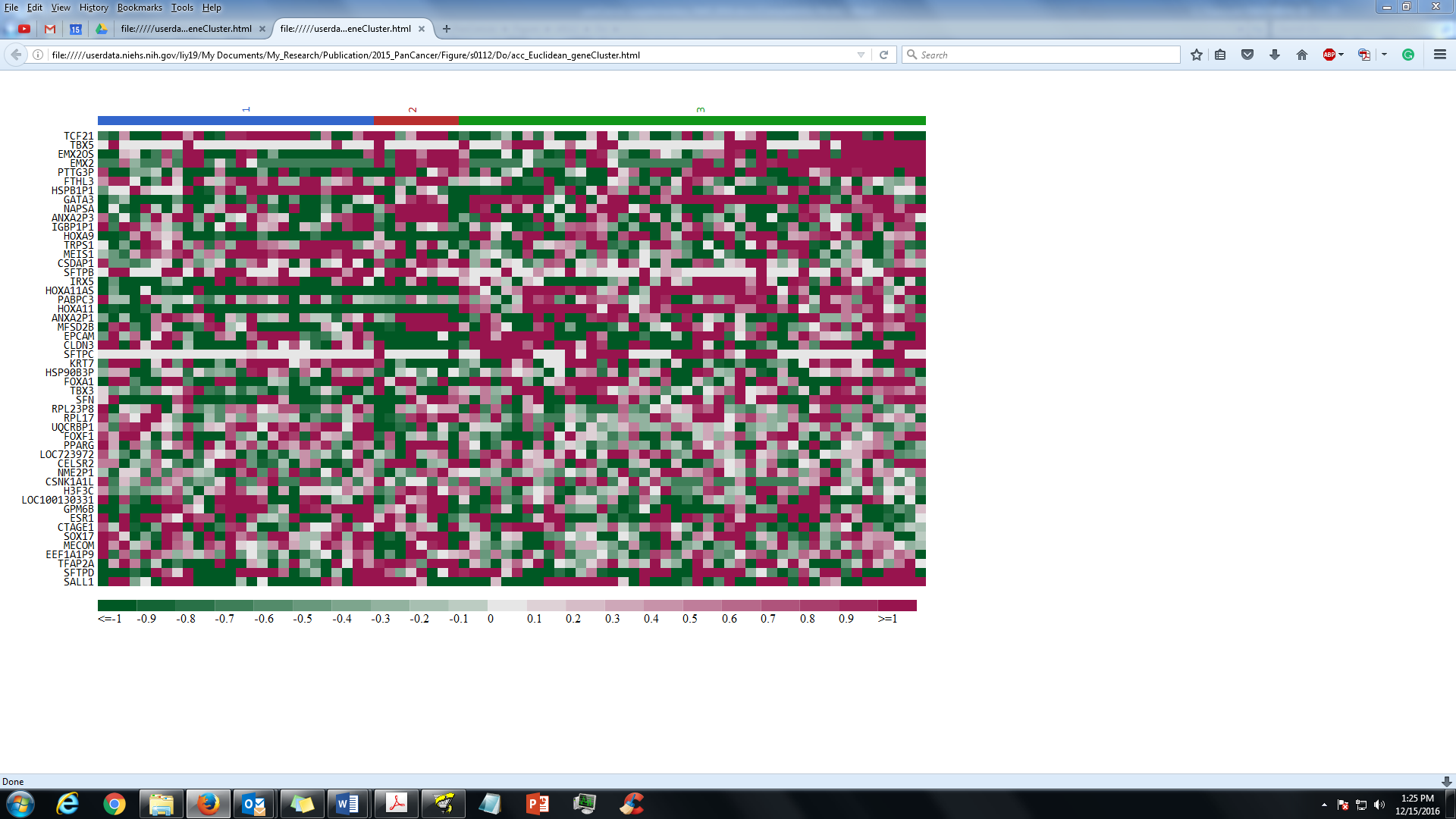


1. **Adrenocortical carcinoma (ACC)**

**
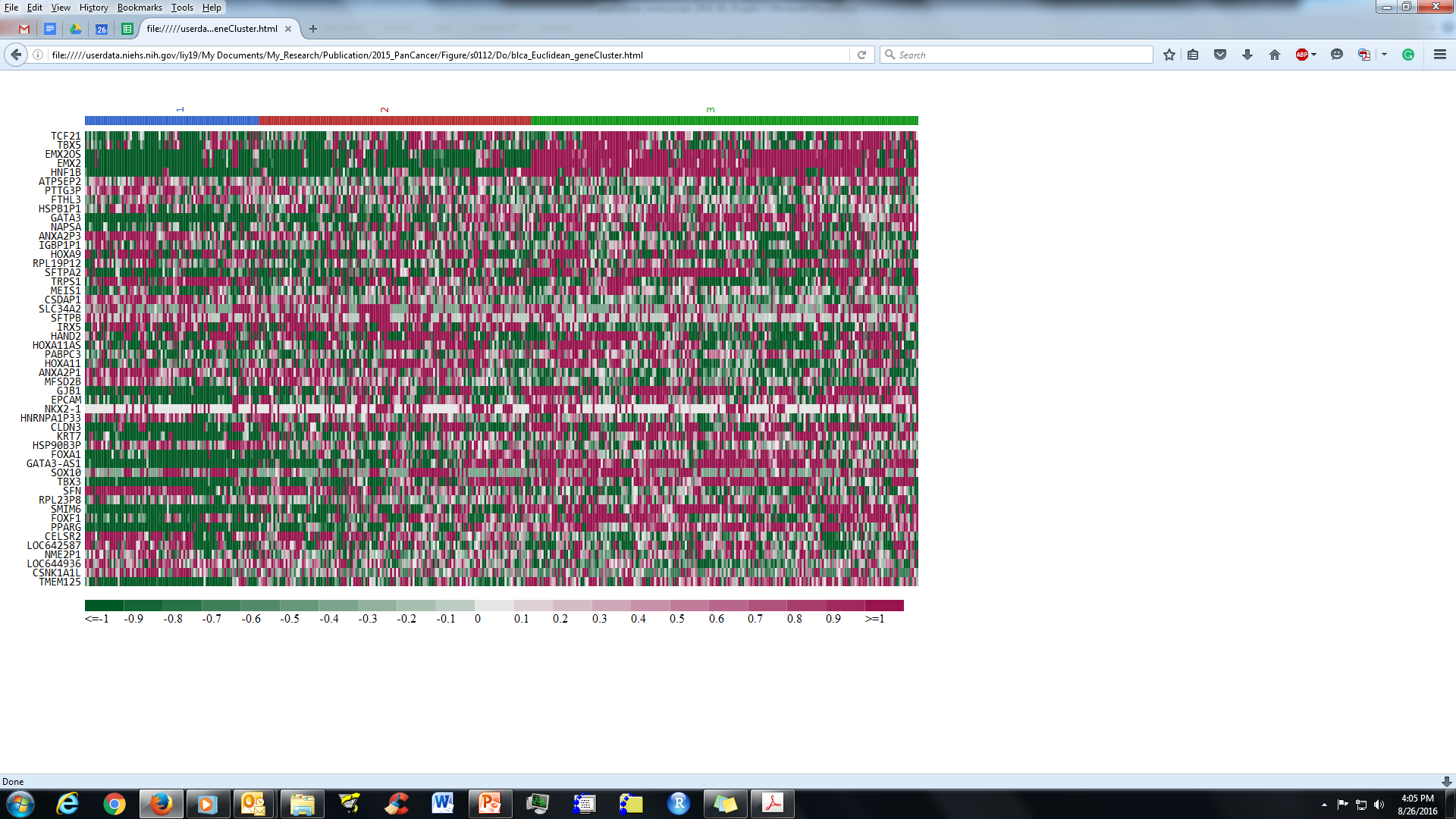
**

1. **Bladder urothelial carcinoma (BLCA)**

**
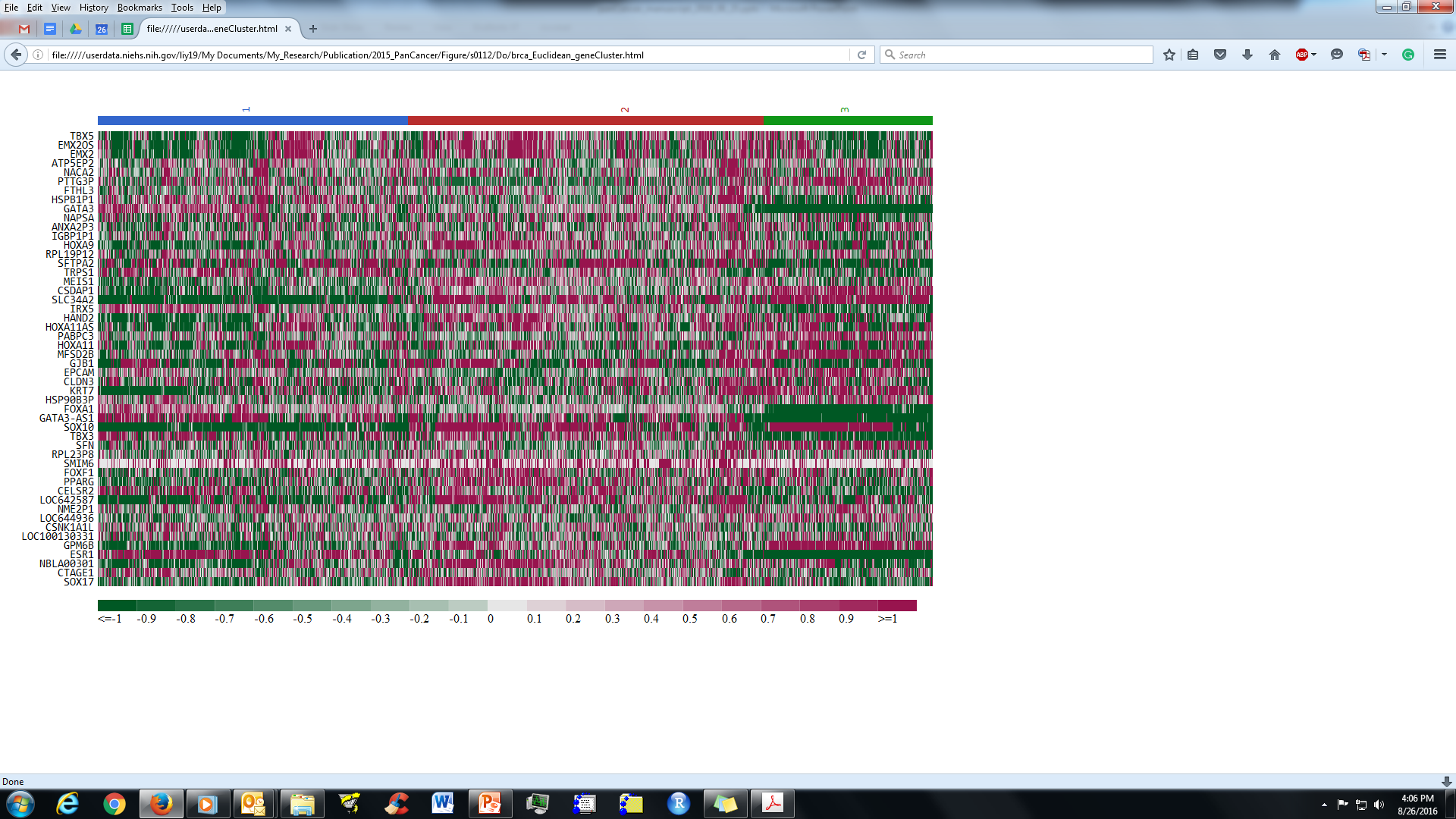
**

1. **Breast invasive carcinoma (BRCA)**

**
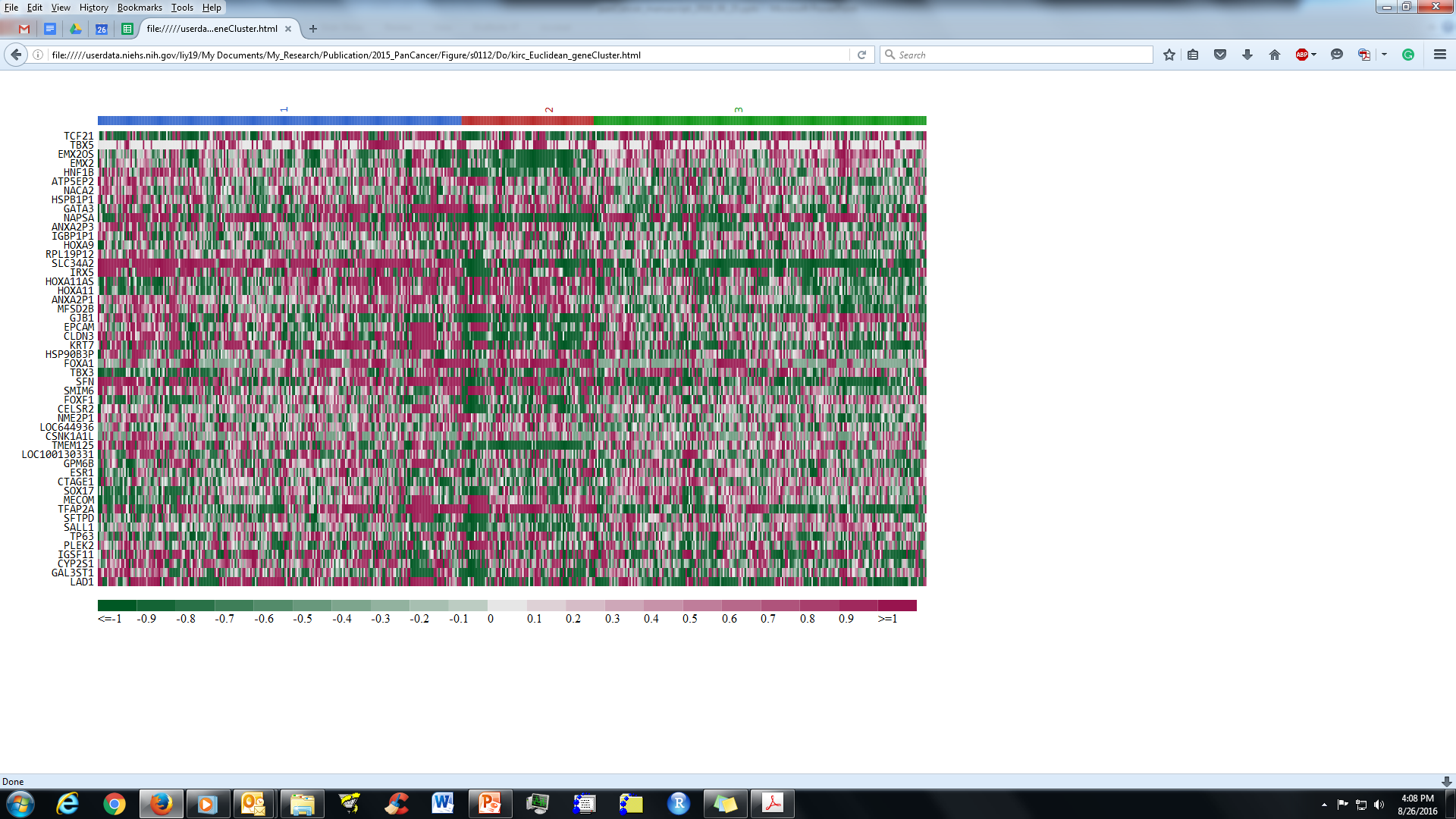
**

1. **Kidney renal clear cell carcinoma (KIRC)**

**
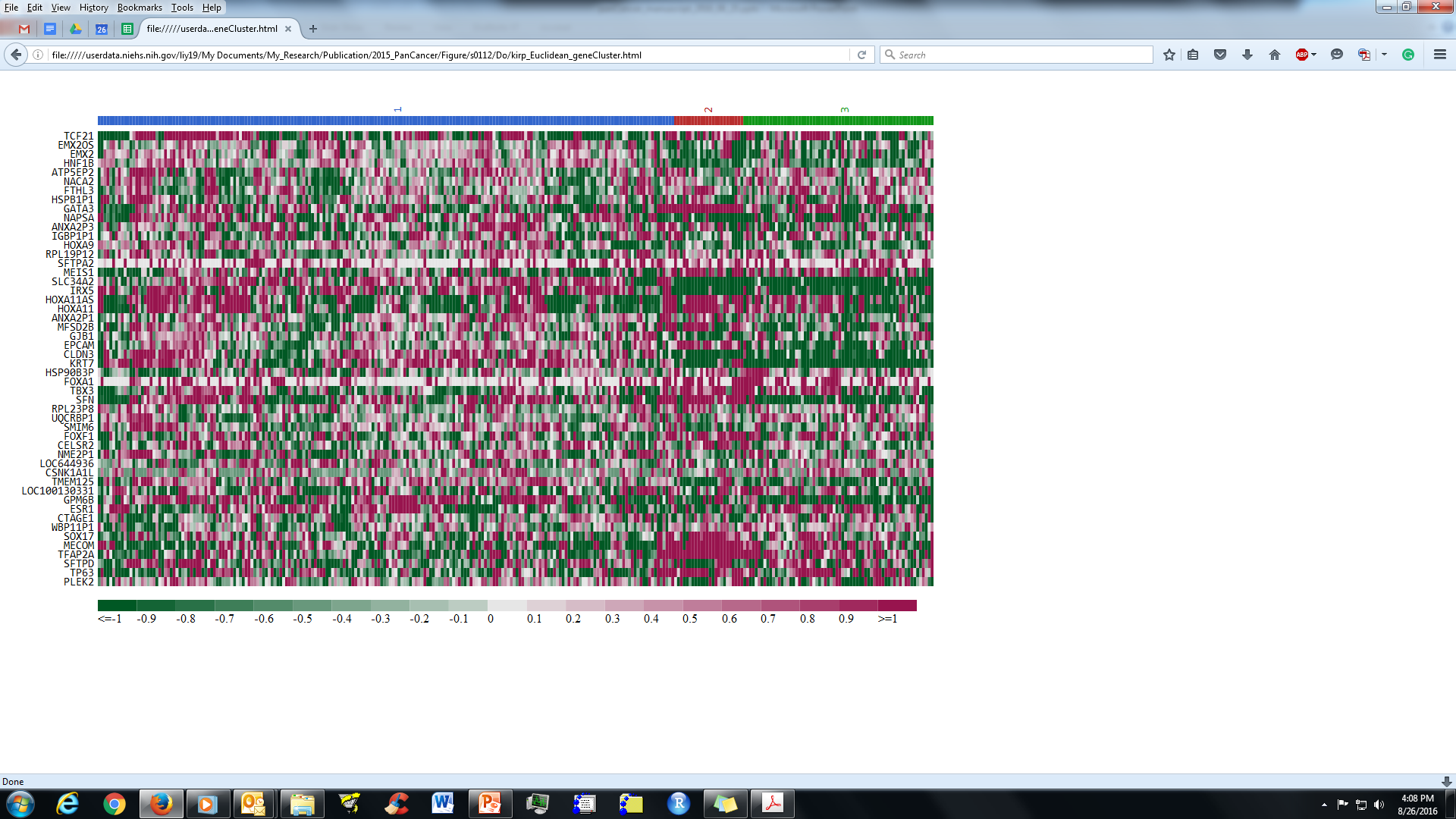
**

1. **Kidney renal papillary cell carcinoma (KIRP)**

**
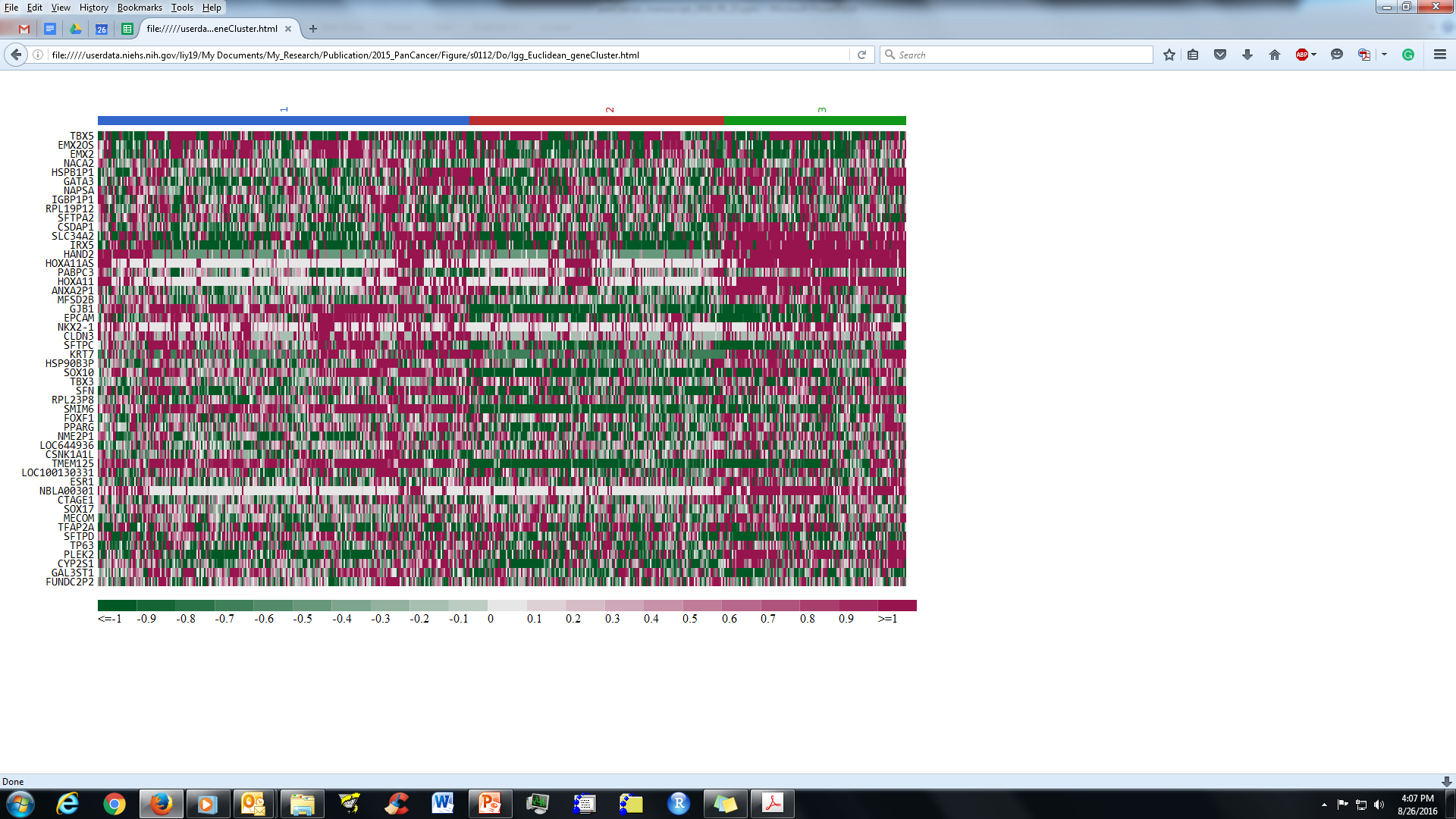
**

1. **Brain lower grade glioma (LGG)**

**
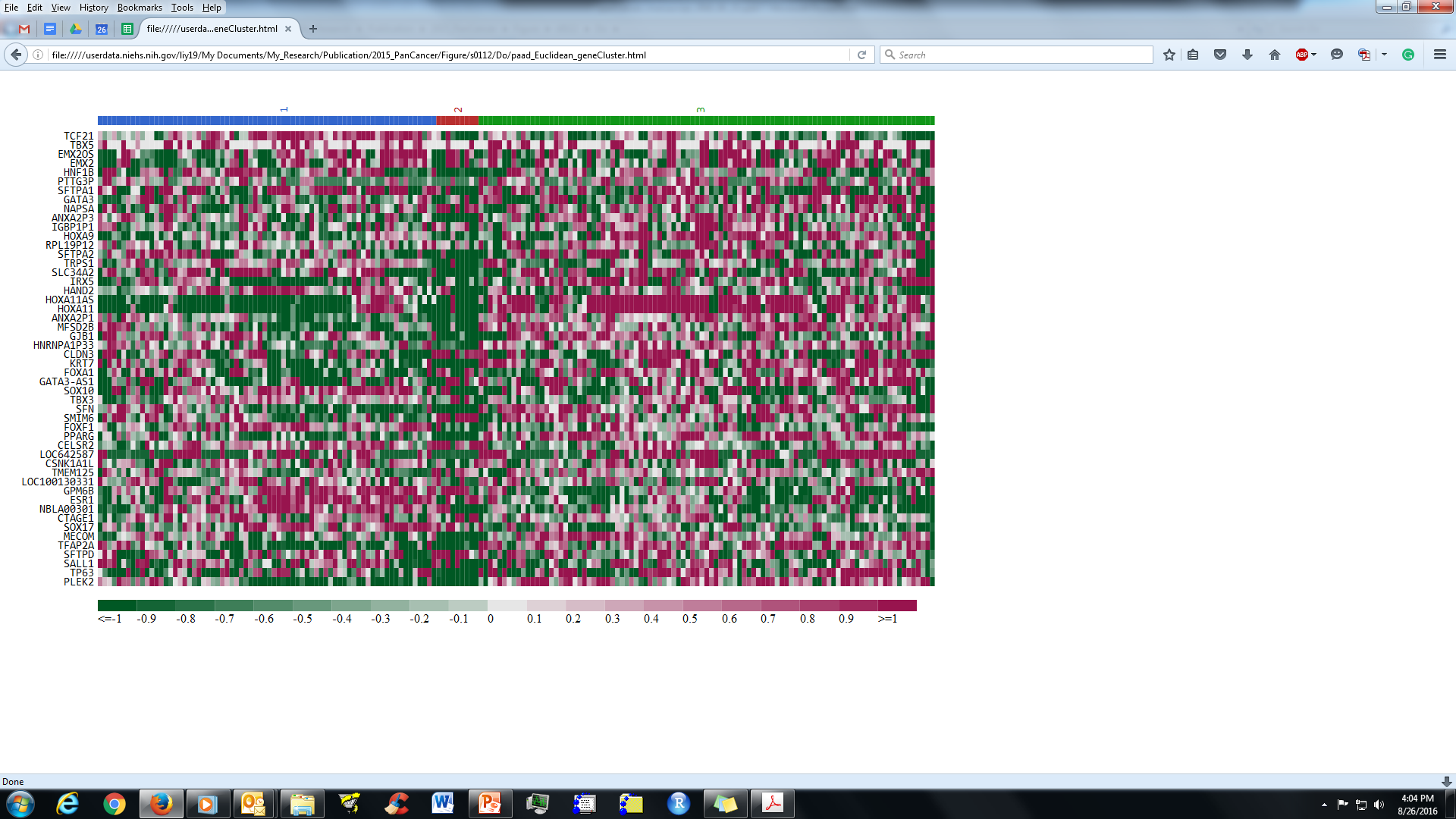
**

1. **Pancreatic adenocarcinoma (PAAD)**

**Figures S2** Heatmap representation of the expression patterns of the top 50 genes across all (a) ACC (b) BLCA, (c) BRCA, (d) KIRC, (e) KIRP, (f) LGG, and (g) PAAD samples. See Figure 3 legend for details. The colors of the horizontal bar represent the subgroups identified by *k*-means clustering analysis.
