# Supplementary material for: A comprehensive genomic pan-cancer classification using The Cancer Genome Atlas gene expression data
Source: BMC Genomics. 2017 Jul 3;18:508. doi: 10.1186/s12864-017-3906-0 (PMC5496318; doi:10.1186/s12864-017-3906-0)
Supplement: Supplementary file 12 — Genes ranked among the top 100 from either females and males. (DOCX 389 kb) [file 12864_2017_3906_MOESM12_ESM.docx]

**Additional file 12: Supplementary Figure S4 for**

**A comprehensive genomic pan-cancer classification using The Cancer Genome Atlas gene expression data**


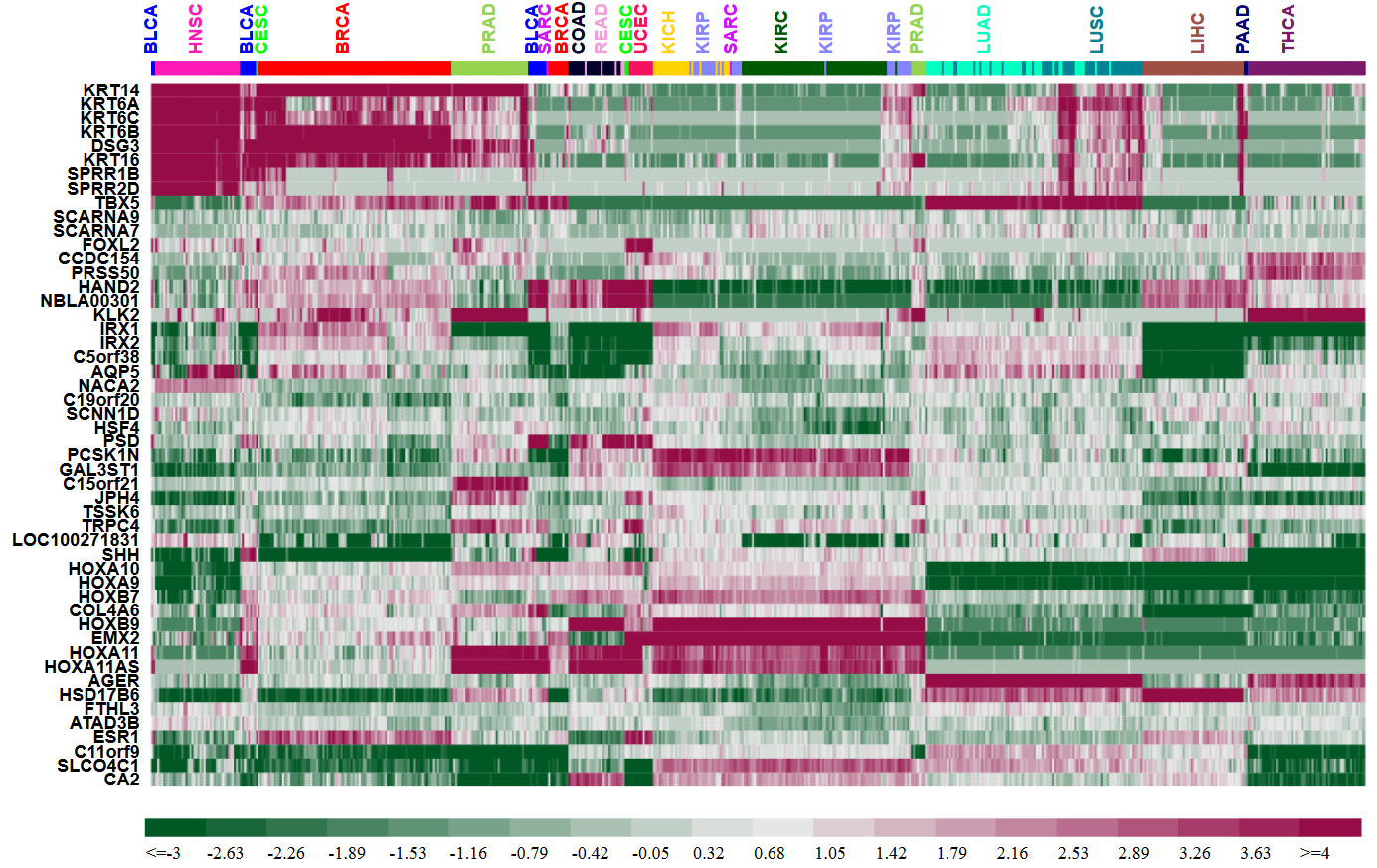


**Figure S4** Heatmap representation of the expression patterns of the top 50 genes selected by GA/KNN across all 602 “normal” samples taken adjacent to tumors from 17 tumor types. Each row (gene) was centered by the median expression value across all samples. A hierarchical clustering analysis was carried out for both samples and genes using the Euclidean distance as the similarity metric.
