# Supplementary material for: A comprehensive genomic pan-cancer classification using The Cancer Genome Atlas gene expression data
Source: BMC Genomics. 2017 Jul 3;18:508. doi: 10.1186/s12864-017-3906-0 (PMC5496318; doi:10.1186/s12864-017-3906-0)
Supplement: Supplementary file 13 — Heatmap representations of the expression patterns of the top genes across all male and female samples. Each row (gene) was centered by the median expression value across all samples. A hierarchical clustering analysis was carried out for both samples and genes using the Euclidean distance as the similarity metric. (DOCX 15 kb) [file 12864_2017_3906_MOESM5_ESM.docx]

**Additional file 5: Methods for**

**A comprehensive genomic pan-cancer classification using The Cancer Genome Atlas gene expression data**

**Statistical analysis**

We updated the official gene symbols for the ~19,000 genes, including 658 pseudogenes, using gene IDs (formerly known as the locus IDs) from the current version of NCBI gene annotation. The enrichment of pseudogenes among the top 50 genes was calculated in R using the hypergeometric distribution. Analysis for differential expression between males and females in 23 tumor types was carried out using the Mann-Whitney-Wilcoxon rank sum test (two sided).

**Data for survival analysis**

TCGA data for each cancer sample includes gene expression levels through RNA-seq along with information on characteristics of both patients (e.g., demographics, vital status at the time of report, treatment regimens, and clinical follow-up) and their tumors (e.g., disease-specific diagnostic/prognostic factors).

The accurate stage of disease at the time of the TCGA biospecimen procurement was often not available. The pathological data such as primary tumor staging information were referenced to the patient’s initial cancer diagnosis, and often there is a lag between the initial cancer diagnosis and TCGA biospecimen procurement, our analysis is based on patients’ survival from the time of TCGA biospecimen procurement to death or last follow-up. Specifically, the “curated post-procurement survival” is calculated as follows, post-procurement survival = days_to_last_contact – days_to_sample_procurement. If a patient has multiple follow-ups, we used the latest lost-to-follow-up date or the earliest death date. In addition, we filtered out patients with negative “post-procurement survival”.

All survival analyses comparing tumor classes were performed using a log-rank test and visualized with Kaplan-Meier survival curves. Though ideally we would like to measure survival time from initial diagnosis to death, we know that patients received their initial diagnoses before TCGA procured their biospecimens; thus, these patients have been at risk for a period before sample procurement. In addition, some patients may die after diagnosis but before their samples could be procured.
