# Supplementary material for: A comprehensive genomic pan-cancer classification using The Cancer Genome Atlas gene expression data
Source: BMC Genomics. 2017 Jul 3;18:508. doi: 10.1186/s12864-017-3906-0 (PMC5496318; doi:10.1186/s12864-017-3906-0)
Supplement: Supplementary file 14 — Boxplot BNC1 expression data in the 23 sex non-specific tumors from males (blue) and females (pink). (DOCX 126 kb) [file 12864_2017_3906_MOESM7_ESM.docx]

**Additional file 7: Figure S3 for**

**A comprehensive genomic pan-cancer classification using The Cancer Genome Atlas gene expression data**

**
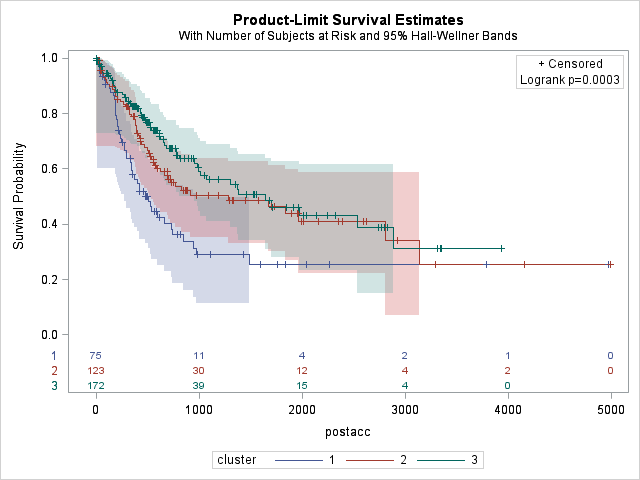
**

1. **Adrenocortical carcinoma (ACC)**

**
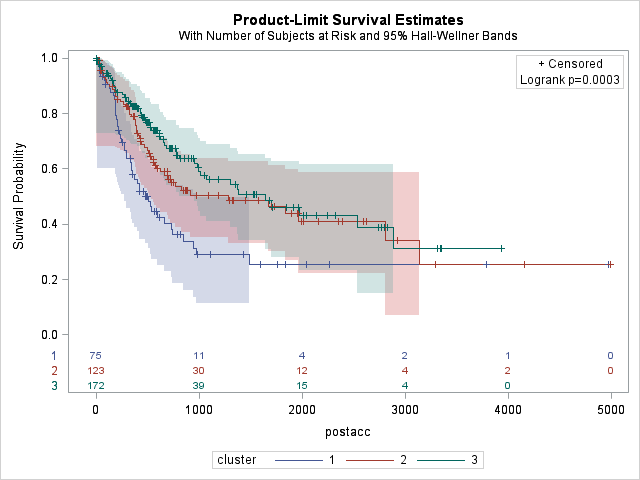
**

1. **Bladder urothelial carcinoma (BLCA)**

**
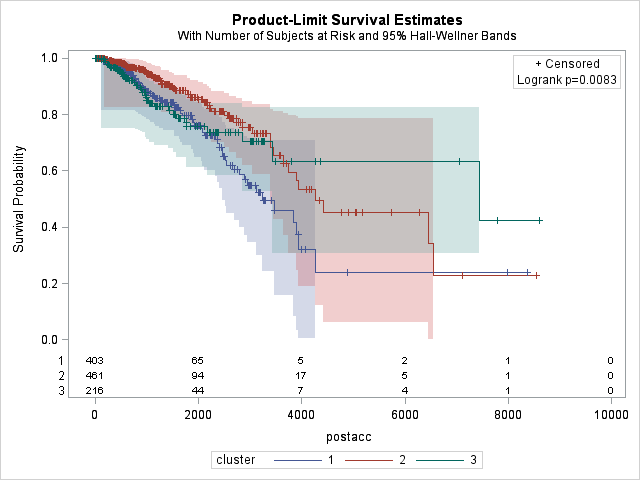
**

1. **Breast invasive carcinoma (BRCA)**

**
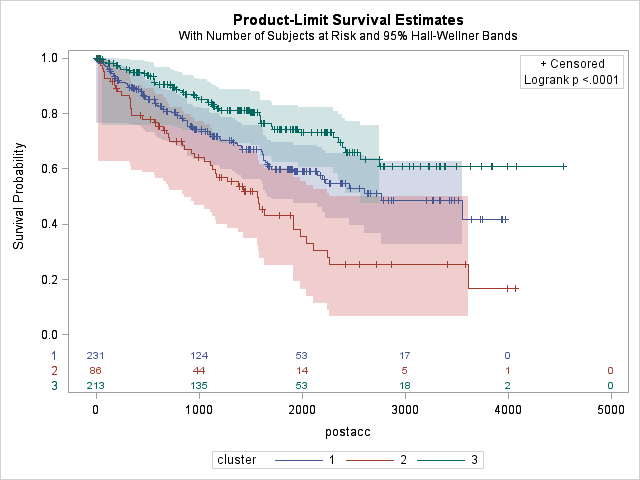
**

1. **Kidney renal clear cell carcinoma (KIRC)**

**
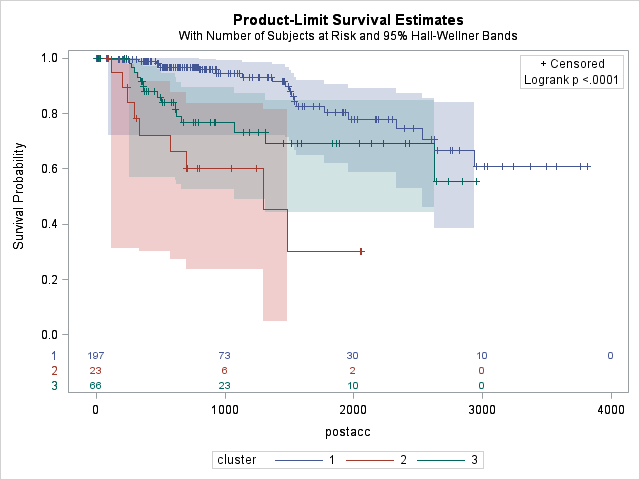
**

1. **Kidney renal papillary cell carcinoma (KIRP)**

**
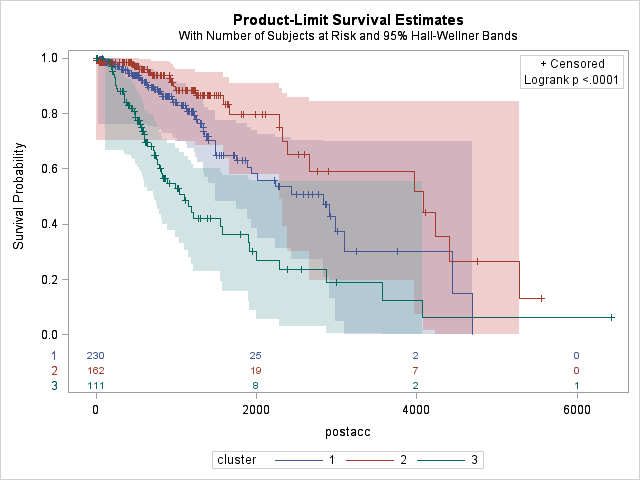
**

1. **Brain lower grade glioma (LGG)**

**
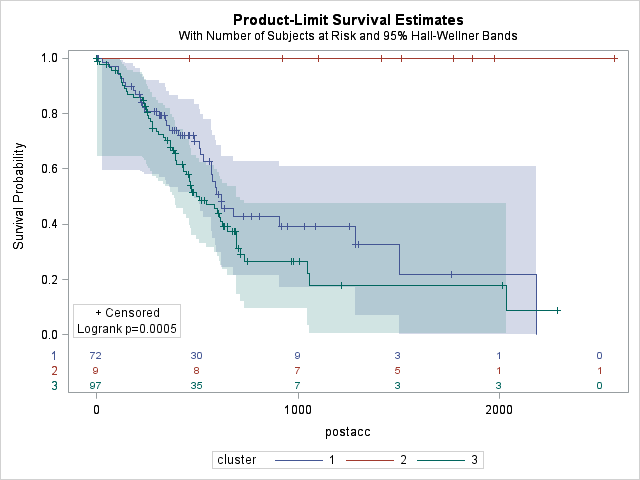
**

1. **Pancreatic agenocarcinoma (PAAD)**

**Figure S3** Post-procurement survival probability for patients in the three subtypes of (a) ACC (b) BLCA, (c) BRCA, (d) KIRC, (e) KIRP, (f) LGG, and (g) PAAD tumors identified by *k*-means analysis based on RNA-seq expression data of the top 50 genes.
