# Supplementary material for: A comprehensive genomic pan-cancer classification using The Cancer Genome Atlas gene expression data
Source: BMC Genomics. 2017 Jul 3;18:508. doi: 10.1186/s12864-017-3906-0 (PMC5496318; doi:10.1186/s12864-017-3906-0)
Supplement: Supplementary file 15 — Mean and median of for πcc values for each tumor type from full female dataset, full male dataset, and the corresponding mean (sd) from the eight “matched” male datasets. (DOCX 283 kb) [file 12864_2017_3906_MOESM15_ESM.docx]

**Additional file 15: Figure S5 for**

**A comprehensive genomic pan-cancer classification using The Cancer Genome Atlas gene expression data**


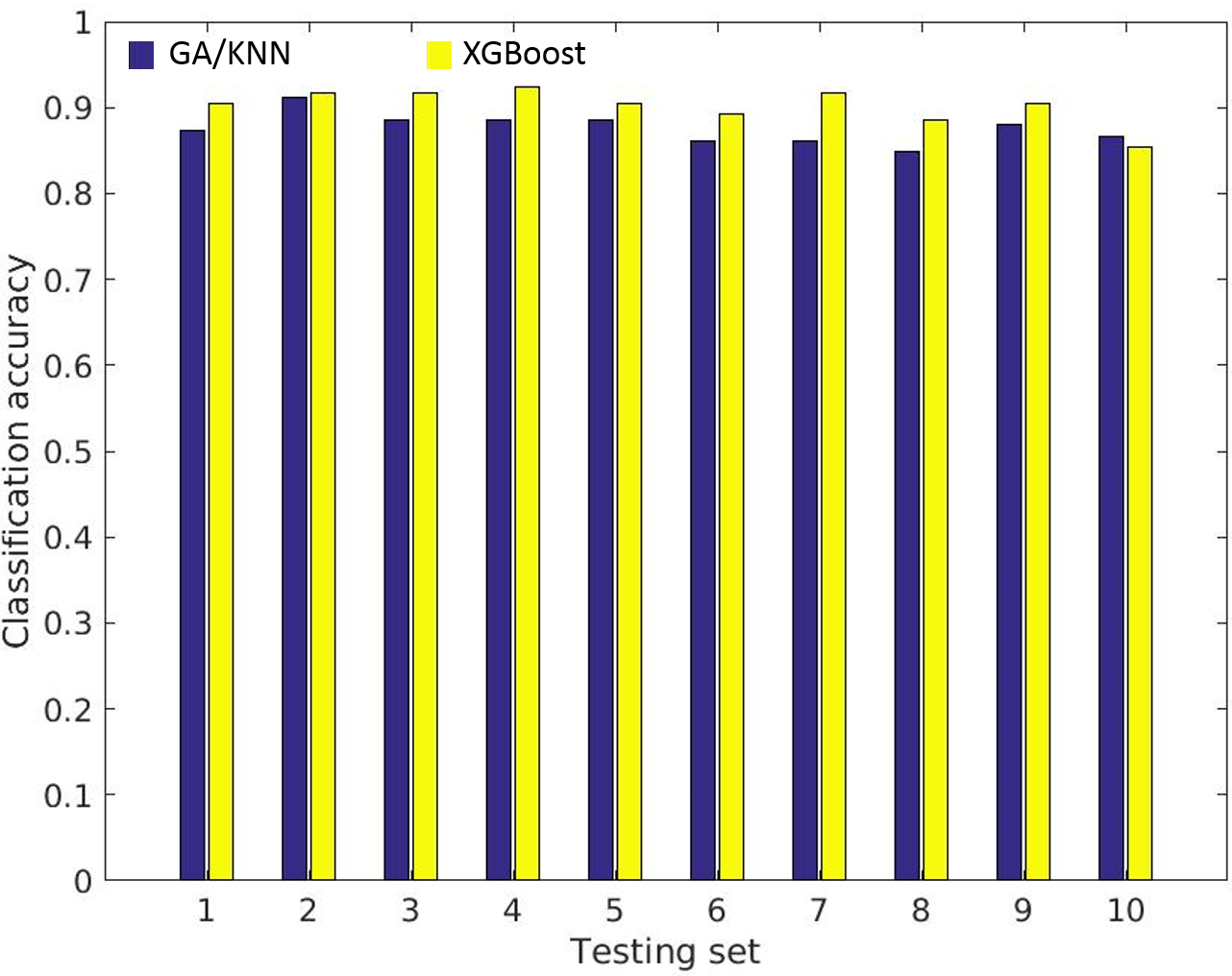


**Figure S5** Classification accuracies between GA/KNN and XGBoost for 10 testing sets.
